# Altered dynamic functional connectivity in adults with rural left-behind experience: a resting-state fMRI study

**DOI:** 10.3389/fpsyt.2026.1900163

**Published:** 2026-08-26

**Authors:** Boyan Cui, Luqing Lan, Changjuan Xie, Siying Liu, Bingbing Ling, Huiru Li, Hao Li, Yin Mo

**Affiliations:** 1 Department of Medical Imaging, The First Affiliated Hospital of Kunming Medical University, Kunming, Yunnan, China; 2 Department of Urology, The First Affiliated Hospital of Kunming Medical University, Kunming, Yunnan, China

**Keywords:** dynamic functional network connectivity (dFNC), early-life social adversity, group independent component analysis (ICA), psychological resilience, resting-state fMRI, rural left-behind experience

## Abstract

**Background:**

Early-life parental separation due to rural labor migration represents a prevalent form of early-life social adversity; however, its neural correlates in adulthood remain insufficiently understood, particularly from the perspective of dynamic brain network organization.

**Methods:**

In this study, resting-state fMRI data were analyzed in adults with left-behind experience (LBE, n = 152) and participants in the non-left-behind comparison group (NLB, n = 145). Group independent component analysis was first used to extract major resting-state networks. Dynamic functional network connectivity was then estimated with a sliding-window approach, and recurrent connectivity configurations were identified using clustering analysis. Group differences in static connectivity, dynamic connectivity states, and temporal properties were examined while accounting for demographic and motion-related covariates. Associations of State 6 temporal properties with LBE duration, psychological resilience, and depressive symptom severity were further explored.

**Results:**

No significant group differences were observed in static functional connectivity or in state-wise connectivity strength. In contrast, significant group differences were identified only in the temporal properties of State 6. Specifically, individuals with LBE exhibited reduced fraction of time (FT) and mean dwell time (MDT) in State 6 after FDR correction, reflecting less frequent and shorter engagement with this connectivity pattern. Among LBE participants who exhibited State 6, longer LBE duration was associated with lower FT in this state after controlling for covariates. Furthermore, MDT in this state was positively associated with optimism scores after controlling for covariates.

**Conclusions:**

These findings suggested that rural left-behind experience was associated with differences in the temporal engagement of a specific dynamic connectivity state in adulthood, despite the absence of detectable group differences in static connectivity or state-wise connectivity strength. The associations of State 6 temporal properties with LBE duration and optimism provided preliminary correlational evidence that dynamic brain-state measures may be related to both characteristics of early-life experience and adaptive capacity.

## 1 Introduction

Early-life social adversity has been widely associated with differences in brain development and mental health outcomes (1, 2). This broad construct encompasses heterogeneous experiences, including family disruption, caregiving deprivation, childhood maltreatment, and prolonged parent–child separation, which may involve partly distinct developmental contexts. Within this broader context, rural left-behind experience (LBE)—arising from prolonged parent–child separation due to labor migration—is highly prevalent in developing regions (3). Previous studies linked this experience to increased vulnerability to internalizing symptoms, including depression and anxiety, particularly during childhood and adolescence (4, 5). Beyond emotional disturbances, previous studies also reported broader psychosocial difficulties among left-behind children, including reduced self-esteem, greater behavioral problems, and impaired social functioning (6, 7). Together, these findings suggested that early parental separation was associated with psychological difficulties during childhood and adolescence; whether related differences remain detectable in adulthood is less well understood.

Although neuroimaging research on early-life social adversity more broadly is substantial, studies specifically examining rural left-behind populations remain relatively limited. Existing studies primarily examined static brain characteristics in left-behind children. Zhao et al. reported increased normalized characteristic path length and altered nodal centralities in frontolimbic, motor, and sensory regions, whereas Wang et al. identified altered amplitude of low-frequency fluctuation (ALFF) and fractional amplitude of low-frequency fluctuation (fALFF) in regions involved in cognitive and emotional processing, including the insula, orbitofrontal cortex, hippocampus, and superior temporal gyrus (8, 9). These findings provided initial evidence of altered brain organization in left-behind children but did not establish whether related differences are detectable in adulthood. More generally, many prior neuroimaging studies of early-life social adversity have emphasized static functional connectivity and regional activity metrics, which reflect time-averaged patterns of brain organization. However, these approaches may not adequately capture the continuously evolving interactions among large-scale brain networks (10). Therefore, it remains unclear whether LBE is associated with differences in brain network organization in adulthood and whether such differences are expressed in the temporal dynamics of brain networks.

Dynamic functional network connectivity (dFNC) offers a framework for examining time-resolved interactions within large-scale brain systems (11, 12). By combining group independent component analysis (ICA), sliding-window estimation, and clustering, dFNC identifies recurring connectivity states and characterizes their temporal properties (13, 14). Temporal characteristics of these states can be quantified using fraction of time (FT; defined as the proportion of sliding windows assigned to a given state), mean dwell time (MDT; the average duration within a state), and number of transitions (NT; the frequency of switching between states) (14). These indices reflect the temporal organization of brain network dynamics, capturing aspects of the stability and flexibility of large-scale systems that may be obscured by time-averaged connectivity measures (14–17). This distinction is conceptually relevant to LBE because early-life social adversity has been associated with differences in large-scale brain network organization, which may also be expressed in the frequency, persistence, and switching of transient connectivity states (2, 10). Consistent with this possibility, childhood trauma was associated with increased network switching and reduced temporal stability of functional brain networks, whereas childhood emotional neglect was linked to longer dwell time in a weakly connected state and fewer state transitions, suggesting reduced functional network flexibility in young adults (18, 19). Psychological resilience reflects adaptive capacity following adversity, whereas depressive symptoms reflect symptom burden; examining their associations with dFNC measures may therefore help clarify the functional relevance of brain network temporal dynamics.

**Figure 1:**
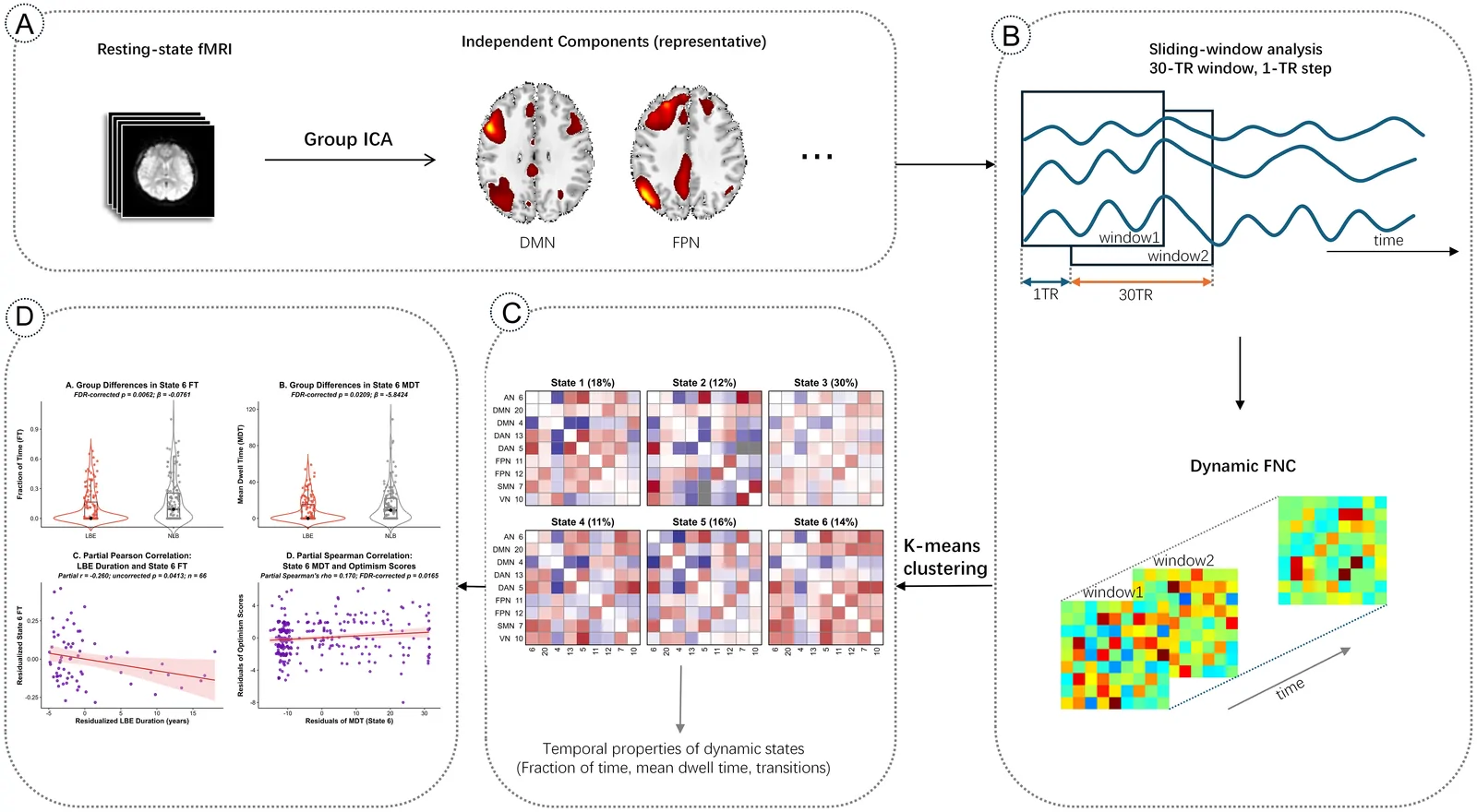
Resting-state fMRI data were preprocessed and analyzed using a group independent component analysis (ICA) framework to identify resting-state networks (RSNs). For dynamic functional network connectivity (dFNC), a sliding-window approach was applied to component time courses to generate time-resolved connectivity matrices, which were clustered using k-means to identify recurring connectivity states. Temporal properties of each state, including fraction of time (FT), mean dwell time (MDT), and number of transitions (NT), were calculated and examined in relation to LBE duration and questionnaire-derived measures. **(A)** rs-fMRI data were decomposed into independent components; **(B)** sliding-window analysis captured temporal variability; **(C)** k-means clustering identified discrete dynamic connectivity states and their temporal properties; and **(D)** statistical analyses assessed group differences and associations with LBE duration and optimism scores.

In the present study, we applied a group ICA-based dFNC framework to examine whether LBE was associated with differences in the dynamic organization of large-scale brain networks in adulthood. Specifically, we addressed two questions: (1) whether individuals with LBE showed differences in temporal properties—including FT, MDT, and NT—of recurring connectivity states compared with the non-left-behind comparison group (NLB); and (2) whether these dynamic measures were related to psychological resilience and depressive symptom severity, which index adaptive capacity and symptom burden, respectively. In an exploratory analysis within the LBE group, we further examined whether FT and MDT in the state showing significant group differences were associated with the duration of LBE. Given that dFNC captures the stability and flexibility of transient brain states, we hypothesized that the LBE group would exhibit differences in temporal characteristics, such as FT and MDT, and that these temporal measures would be associated with individual differences in psychological resilience and depressive symptom severity. By examining these questions in adulthood, this study aimed to extend current understanding of the association between early parental separation and adult brain network dynamics. A schematic overview of the analytical pipeline is provided in **Figure 1**.

## 2 Methods

### 2.1 Participants

Participants aged 20–40 years were recruited from rural areas in Yunnan Province. All participants had a rural household registration and had resided in rural areas for more than 16 years, to minimize the potential influence of urban–rural environmental differences on brain function. To reduce potential confounding effects related to cultural and environmental diversity, only participants of Han Chinese ethnicity were included, thereby minimizing the influence of ethnic heterogeneity.

Participants were divided into two groups based exclusively on rural left-behind experience. Participants in the LBE group were defined as those who experienced parental migration (one or both parents) for at least 2 years before the age of 17. Participants who did not meet this criterion were assigned to the NLB group. Other forms of early-life social adversity, social support, and caregiving quality were not incorporated into group classification. LBE duration was assessed using a self-report questionnaire and was defined as the cumulative duration before the age of 17 during which both parents were simultaneously absent from home for migrant work.

Inclusion criteria were as follows: (1) age between 20 and 40 years; (2) rural household registration with at least 16 years of residence in rural areas; (3) right-handedness; and (4) no history of neurological or psychiatric disorders. Exclusion criteria included: (1) history of substance abuse; (2) severe head injury; and (3) contraindications to MRI scanning.

A total of 301 participants were initially recruited for this study. All participants were physically healthy with normal development at the time of assessment. Participants with excessive head motion during fMRI scanning were excluded (n = 4), defined as maximum head motion exceeding 3 mm in translation or 3° in rotation. After applying the exclusion criteria, the final sample consisted of 297 participants, including 152 participants in the LBE group and 145 participants in the NLB group. This study was approved by the Ethics Committee of The First Affiliated Hospital of Kunming Medical University. All procedures were conducted in accordance with the ethical standards of the institutional research committee. All participants provided written informed consent prior to participation. **Table 1** summarizes the demographic characteristics and questionnaire scores of the participants.

**Table 1:** Demographic characteristics and questionnaire scores of the LBE and NLB groups.

Variable	LBE group (n = 152)	NLB group (n = 145)	Test statistic	p value
Age, years	24.66 ± 3.35	24.54 ± 2.12	t = 0.371	0.711
Male, n (%)	68 (44.7%)	61 (42.1%)	χ² = 0.120	0.729
Education, years	16.77 ± 1.83	17.71 ± 1.65	t = −4.653	**<0.001**
Mean FD (mm)	0.13 ± 0.06	0.13 ± 0.07	t = 0.004	0.997
SDS score	37.03 ± 7.08	34.06 ± 6.83	t = 3.324	**0.001**
CD-RISC score	61.76 ± 12.26	65.71 ± 12.63	t = −2.670	**0.008**
Optimism score	9.74 ± 2.21	10.52 ± 2.09	t = −2.828	**0.005**
Strength score	21.75 ± 4.33	22.62 ± 4.29	t = −1.739	0.083
Tenacity score	30.34 ± 7.08	32.70 ± 7.46	t = −2.670	**0.008**

Data are presented as mean ± SD or n (%). Group differences were assessed using Welch’s independent-samples t-tests for continuous variables and a chi-square test for sex. FD, framewise displacement; SDS, Self-Rating Depression Scale; CD-RISC, Connor–Davidson Resilience Scale. Bold values indicate statistically significant p values.

### 2.2 Data acquisition and preprocessing

MRI data were acquired using a 3.0 Tesla GE Discovery MR750W scanner (GE Healthcare, Milwaukee, WI, USA). During the resting-state functional MRI (rs-fMRI) scan, participants were instructed to lie still with their eyes closed, remain awake, and avoid engaging in specific thoughts.

Functional images were obtained using a gradient-echo echo-planar imaging (EPI) sequence with the following parameters: repetition time (TR) = 2000 ms, echo time (TE) = 30 ms, slice thickness = 4 mm, inter-slice gap = 0.6 mm, number of slices = 33, field of view (FOV) = 220 × 220 mm², number of excitations (NEX) = 1, and flip angle = 90°. Images were acquired in the axial plane, yielding 160 volumes per participant. High-resolution T1-weighted structural images were acquired using a 3D BRAVO sequence with the following parameters: repetition time (TR) = 8.56 ms, echo time (TE) = 3.22 ms, inversion time (TI) = 450 ms, flip angle = 12°, field of view = 256 × 256 mm², matrix size = 256 × 256, slice thickness = 1 mm with no gap, and voxel size = 1 × 1 × 1 mm³.

The preprocessing of fMRI data was performed using the Data Processing and Analysis of Brain Imaging toolbox (DPABI v6.0, https://rfmri.org/dpabi) (20), which is based on Statistical Parametric Mapping (SPM12, version 7219, https://www.fil.ion.ucl.ac.uk/spm/software/spm12/), and implemented in MATLAB R2021b (The MathWorks, Inc., Natick, MA, USA). The first 10 volumes of each functional time series were discarded to allow for signal equilibration and participant adaptation. The remaining images were corrected for slice timing and realigned to account for head motion.

Functional images were then co-registered to the corresponding T1-weighted structural images. Structural images were segmented, and spatial normalization to the standard Montreal Neurological Institute (MNI) space was performed using the DARTEL approach. The normalized functional images were resampled to 3 × 3 × 3 mm³ voxels. Finally, spatial smoothing was applied using a Gaussian kernel with a full width at half maximum (FWHM) of 4 mm.

### 2.3 Assessment of psychological resilience and depressive symptoms

Psychological resilience was assessed using the Connor–Davidson Resilience Scale (CD-RISC), which has been validated in Chinese populations (21). The Chinese version of the scale comprises multiple dimensions, including optimism, strength, and tenacity, and is rated using a five-point Likert scale. Higher scores indicate greater levels of psychological resilience.

Depressive symptoms were assessed using the Self-Rating Depression Scale (SDS) (22), a widely used self-report instrument for evaluating the severity of depressive symptoms in both clinical and non-clinical populations. The SDS consists of 20 items rated on a four-point Likert scale, reflecting the frequency of depressive symptoms experienced by the individual. The total score is calculated by summing all items, with higher scores indicating greater severity of depressive symptoms. The SDS and CD-RISC were used to assess depressive symptom burden and psychological resilience, respectively, and were not used for group classification. In the present study, the SDS total score, CD-RISC total score, and the optimism, strength, and tenacity subscale scores were extracted for subsequent correlation analyses. These questionnaire scores are presented in **Table 1**.

### 2.4 Group ICA and identification of RSNs

Group independent component analysis (ICA) was performed using the GIFT toolbox (version 4.0.4.7, https://trendscenter.org/software/gift/). The optimal number of independent components (ICs) was estimated using the minimum description length (MDL) criterion. Based on these results, 20 ICs were selected for subsequent analysis.

Data dimensionality was first reduced using principal component analysis (PCA), followed by group independent component analysis (ICA) implemented with the Infomax algorithm. To ensure the stability and reliability of the decomposition, ICASSO was performed with 100 repetitions, in which the ICA algorithm was repeatedly applied and the resulting components were clustered based on their similarity. Cluster quality and stability were assessed using the stability index (Iq), with values approaching 1 indicating highly reliable components.

**Figure 2:**
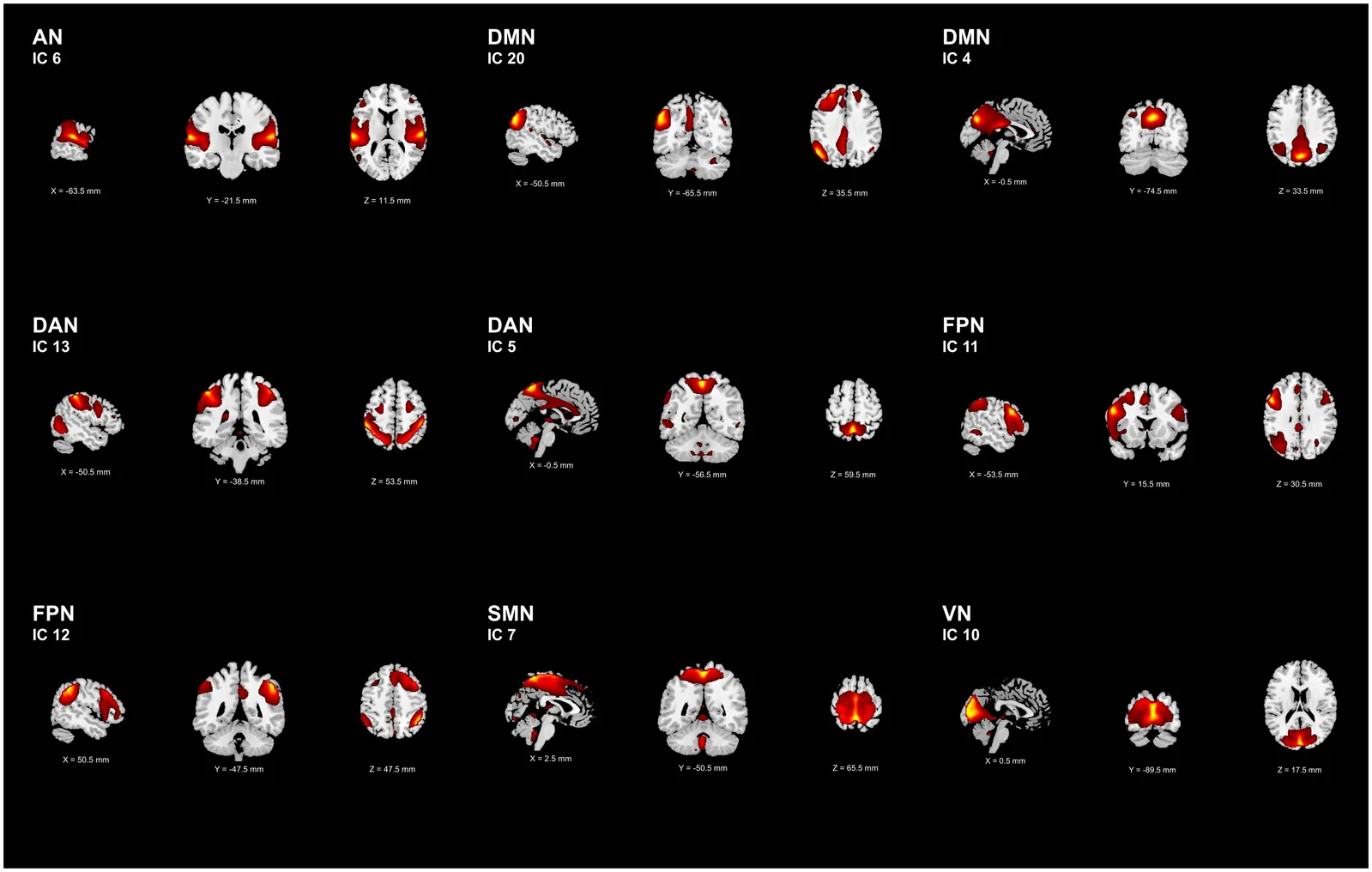
Spatial maps of the identified resting-state networks (RSNs) derived from group independent component analysis. Six canonical RSNs were identified, including the auditory network (AN), default mode network (DMN), dorsal attention network (DAN), frontoparietal network (FPN), somatomotor network (SMN), and visual network (VN). Some networks were represented by multiple independent components (ICs), reflecting spatially distributed subnetworks within the same functional system. Each network is shown with its corresponding IC, with IC numbers indicated above each panel. The displayed slices represent peak activations in MNI space, thresholded at Z > 1.0 for visualization. Warmer colors indicate higher activation strength.

The number of independent components was set to 20 according to the minimum description length (MDL) criterion. The ICASSO results demonstrated high clustering stability across components, indicating robust and reproducible decomposition. Subject-specific spatial maps and time courses were subsequently reconstructed using the GICA back-reconstruction approach, and spatial maps were standardized to Z-scores for further analysis. The resulting components were identified as resting-state networks (RSNs) based on their spatial patterns and consistency with previous literature. In addition, components were selected according to quantitative criteria derived from spatial multiple regression. Components with regression coefficients greater than 0.1 were considered meaningful RSNs. Visual inspection was further applied as a complementary step to confirm the anatomical plausibility of each component. A total of six canonical RSNs were identified, including the auditory network (AN), default mode network (DMN), dorsal attention network (DAN), frontoparietal network (FPN), somatomotor network (SMN), and visual network (VN). Components associated with artifacts (e.g., white matter, cerebrospinal fluid, or motion-related signals) were excluded. Among the 20 ICs, nine components were identified as meaningful RSN-related subnetworks, while the remaining components were classified as noise and excluded from further analysis. The templates for these RSNs, along with the corresponding independent components showing the highest spatial correlations, are presented in **Figure 2**.

### 2.5 Static functional network connectivity

Static functional network connectivity (sFNC) was estimated using the GIFT Mancovan toolbox. For each subject, pairwise Pearson correlations were computed between the time courses of the selected independent components, and the resulting connectivity matrices were transformed using Fisher’s Z transformation. Group differences were examined using a multivariate analysis of covariance (MANCOVA), with group included as the factor of interest and age, sex, years of education, and mean FD entered as covariates. For complete edge-wise analyses, covariate-adjusted general linear models were additionally applied to all 36 sFNC edges using the same covariates, with Benjamini–Hochberg FDR correction across the 36 edges.

### 2.6 Dynamic functional network connectivity analysis

Dynamic functional network connectivity (dFNC) was estimated using a sliding-window approach with a tapered window (30 TRs, step size = 1 TR). This parameter combination has been widely used in previous dFNC studies (23, 24), providing a suitable trade-off between temporal resolution and estimation reliability. Functional connectivity was estimated using a sparsity-constrained inverse covariance method with L1 regularization (LASSO) to promote sparsity. The resulting connectivity matrices were transformed using Fisher’s Z transformation.

Clustering analysis was conducted using the k-means algorithm with squared Euclidean distance. The procedure was repeated 150 times with different initializations, and each run allowed up to 500 iterations to ensure solution stability. Candidate solutions from K = 2 to K = 10 were evaluated using the elbow criterion, silhouette coefficient, gap statistic, and repeated split-half reproducibility analysis. Based on the convergence of these complementary criteria and the parsimony of the solution, K = 6 was retained (see **Supplementary Methods**; **Supplementary Figure 1**).

Group differences in dFNC connectivity strength within each state were assessed using two-sample t-tests implemented in the GIFT toolbox. FDR correction for multiple comparisons was applied using the Benjamini–Hochberg procedure (see Section 2.7 for details). For complete edge-wise analyses, covariate-adjusted general linear models were additionally applied to all 36 dFNC edges within each state, with age, sex, years of education, and mean FD entered as covariates. State-specific analyses included participants with an available subject-level connectivity pattern for the corresponding state, and FDR correction was performed separately across the 36 edges within each state. Temporal properties of dFNC were further calculated, including fraction of time (FT; proportion of time spent in each state), mean dwell time (MDT; average duration within a state), and number of transitions (NT; frequency of switching between states). Additional validation analyses assessed within-state consistency, cross-subject reproducibility, between-state separation, the network-level characteristics of State 6, the potential influence of unequal group contributions on the pooled State 6 centroid, and sensitivity to a shorter 20-TR window; full procedures are provided in the **Supplementary Methods**.

### 2.7 Statistical analysis

Statistical analyses were performed using R version 4.5.3 (R Foundation for Statistical Computing, Vienna, Austria). Continuous variables were presented as mean ± standard deviation (SD), and categorical variables as counts and percentages. Between-group comparisons for continuous variables were conducted using Welch’s t-tests, while categorical variables were analyzed using chi-square tests.

FT and MDT in all six dFNC states and the overall number of transitions (NT) were compared between groups using general linear models (GLM). Group was included as the main independent variable, with age, sex, years of education, and mean framewise displacement (FD) as covariates. The group coefficient represented LBE minus NLB. Regression coefficients, *t* values, and *p* values for the group effect were extracted from each model. Standard errors, 95% confidence intervals, and partial R² values were additionally calculated for the group effects.

Partial Spearman correlation analyses were performed in the full sample (n = 297) to examine the associations of FT and MDT in State 6 with five questionnaire-derived measures, including SDS, CD-RISC total, optimism, strength, and tenacity scores, while controlling for age, sex, years of education, and mean framewise displacement (FD). Participants who did not exhibit State 6 during the scan were retained in these analyses, with FT and MDT coded as 0. Benjamini–Hochberg false discovery rate (FDR) correction was applied separately within each family of tests: (1) across the six states for each temporal metric in the group comparisons, and (2) across the five questionnaire-derived measures separately for each State 6 temporal metric (FT and MDT) in the correlation analyses. No correction for multiple comparisons was applied to the descriptive between-group comparisons of demographic variables and questionnaire scores presented in **Table 1**.

Within the LBE group, exploratory partial Pearson correlation analyses were performed to examine the associations of the duration of simultaneous parental absence with FT and MDT in State 6. These analyses were restricted to LBE participants with nonzero State 6 occupancy (FT > 0; n = 66) and controlled for age, sex, years of education, and mean FD. The same restricted sample was used for both the FT and MDT analyses. Two-sided uncorrected p values were reported for these exploratory analyses. No observations were excluded from the primary analyses on the basis of FT or MDT values. To assess the potential influence of extreme observations, outliers for State 6 FT and MDT were identified separately within the LBE and NLB groups using the 1.5-interquartile-range criterion, defined as values below Q1 − 1.5 × IQR or above Q3 + 1.5 × IQR. For each metric, the original covariate-adjusted general linear model was repeated after excluding metric-specific outliers, and FDR correction was recalculated across the six states for the corresponding temporal metric in the reduced sample.

## 3 Results

### 3.1 Participants

**Table 1** summarizes the demographic characteristics and questionnaire scores of the participants. Questionnaire assessments included depressive symptoms measured by the Self-Rating Depression Scale (SDS) and psychological resilience assessed by the Connor–Davidson Resilience Scale (CD-RISC). The CD-RISC comprises the subdimensions of tenacity, strength, and optimism.

No significant group differences were observed in age, sex distribution, or mean FD (all p > 0.05), indicating good comparability between the LBE and NLB groups. However, the LBE group had significantly fewer years of education than the NLB group (p < 0.001).

Regarding questionnaire scores, the LBE group showed significantly higher SDS scores and lower CD-RISC total and optimism scores compared with the NLB group (all p < 0.01, uncorrected, reported for descriptive purposes). In addition, tenacity scores were significantly lower in the LBE group, whereas no significant group difference was observed for the strength subscale.

### 3.2 Static functional network connectivity analysis

Static functional network connectivity (sFNC) analysis revealed no significant group differences between the LBE and NLB groups (all FDR-corrected p > 0.05; **Supplementary Table 15**).

### 3.3 Dynamic functional network connectivity analysis

**Figure 3:**
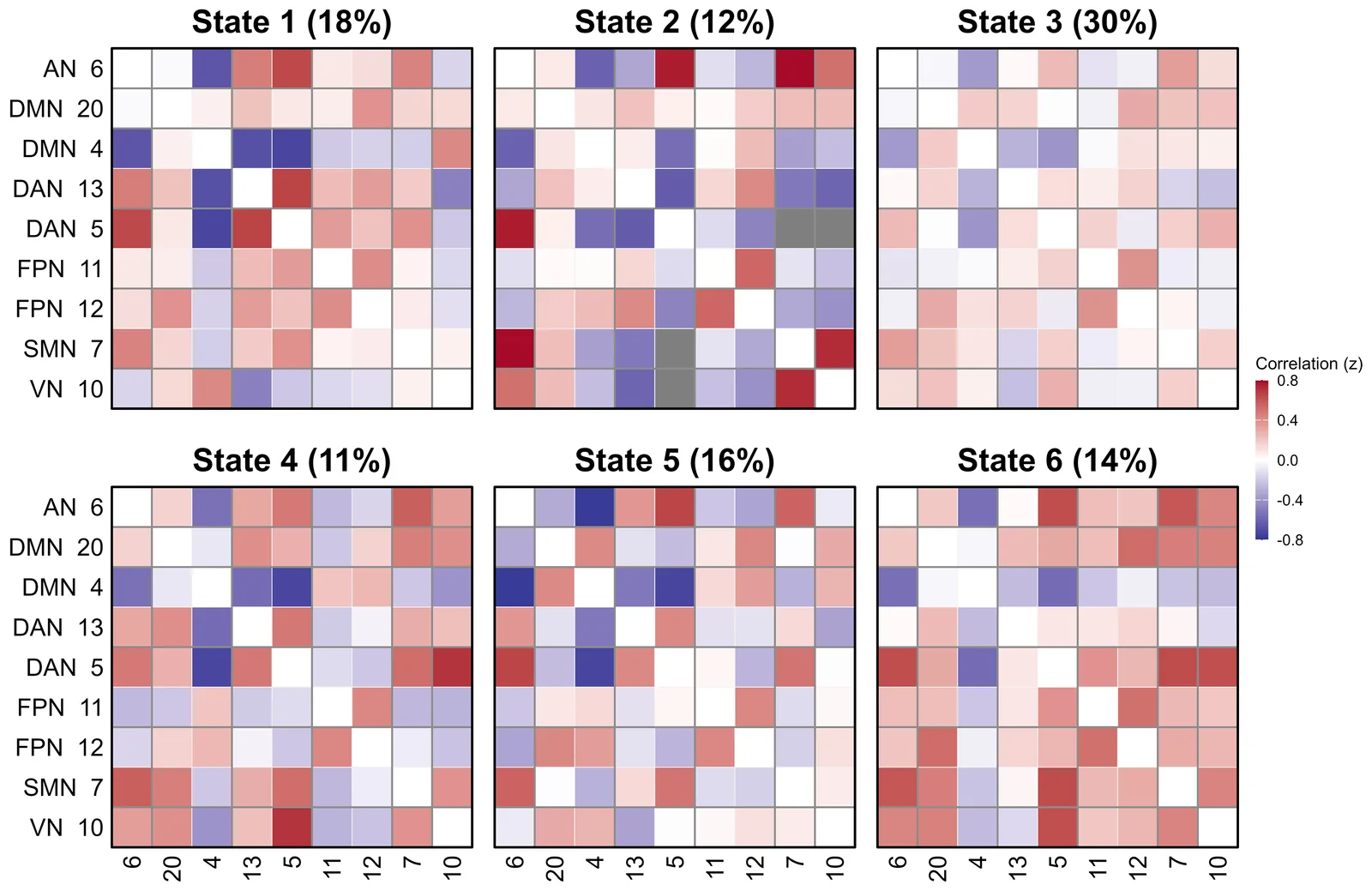
Dynamic functional network connectivity (dFNC) matrices for the six recurring connectivity states identified by k-means clustering. Both axes represent the selected independent components grouped into canonical resting-state networks (RSNs). Connectivity strength is expressed as Fisher z-transformed correlation coefficients, with warm colors indicating positive connectivity and cool colors indicating negative connectivity. All matrices are displayed using a common color scale (Z = −0.8 to 0.8) to facilitate direct comparison across states.

Using a sliding-window approach followed by k-means clustering, six recurring dFNC states were identified across all participants (**Figure 3**), with FT values of 18%, 12%, 30%, 11%, 16%, and 14%, respectively. Complementary validation analyses showed that the elbow criterion, mean silhouette coefficient, and repeated split-half reproducibility supported K = 6, whereas the gap statistic favored K = 8. Based on the convergence of three complementary criteria and the parsimony of the solution, K = 6 was retained (**Supplementary Figure 1**).

State 1 exhibited a moderate connectivity pattern comprising both positive and negative couplings. State 2 showed a more heterogeneous configuration, with relatively stronger within-network coupling in sensory-related networks. State 3, the most prevalent state, was characterized by generally weak and near-zero connectivity. State 4 also exhibited a heterogeneous sensory-related configuration, with relatively stronger within-network coupling. State 5 showed a moderate mixed pattern of positive and negative couplings, broadly resembling State 1. State 6 was characterized by stronger coupling among the somatomotor, auditory, and frontoparietal networks, together with relatively weaker or negative connectivity involving the default mode network.

State 6 showed moderate-to-high within-state consistency (mean window-to-centroid r = 0.661, SD = 0.117) and cross-subject reproducibility (mean subject-to-centroid r = 0.700, SD = 0.113). Pairwise centroid correlations and Euclidean distances are reported in **Supplementary Tables 3–S4**. Quantitative network-level comparisons further showed significantly stronger positive coupling in State 6 for AN–FPN, DMN–SMN, AN–VN, DAN–FPN, DAN–SMN, DAN–VN, FPN–SMN, FPN–VN, and SMN–VN relative to the mean of the other five states after FDR correction (**Supplementary Figure 5**; **Supplementary Table 8**).

Although the LBE group contributed fewer State 6 windows than the NLB group, the LBE- and NLB-specific centroids were highly correlated (r = 0.986), and the equal-group-weighted centroid was nearly identical to the pooled centroid (r = 0.9998), indicating that unequal group contributions did not materially alter the State 6 centroid (**Supplementary Table 6**).

### 3.4 Group differences in state-wise dFNC connectivity strength

Two-sample t-tests were conducted to assess group differences in dFNC connectivity strength across states. No significant differences in connectivity strength were observed between the LBE and NLB groups in any of the six states after FDR correction (all FDR-corrected p > 0.05). Complete covariate-adjusted edge-wise analyses likewise showed that no state-specific dFNC edge survived FDR correction (**Supplementary Table 16**).

### 3.5 Temporal properties of dFNC states

**Figure 4:**
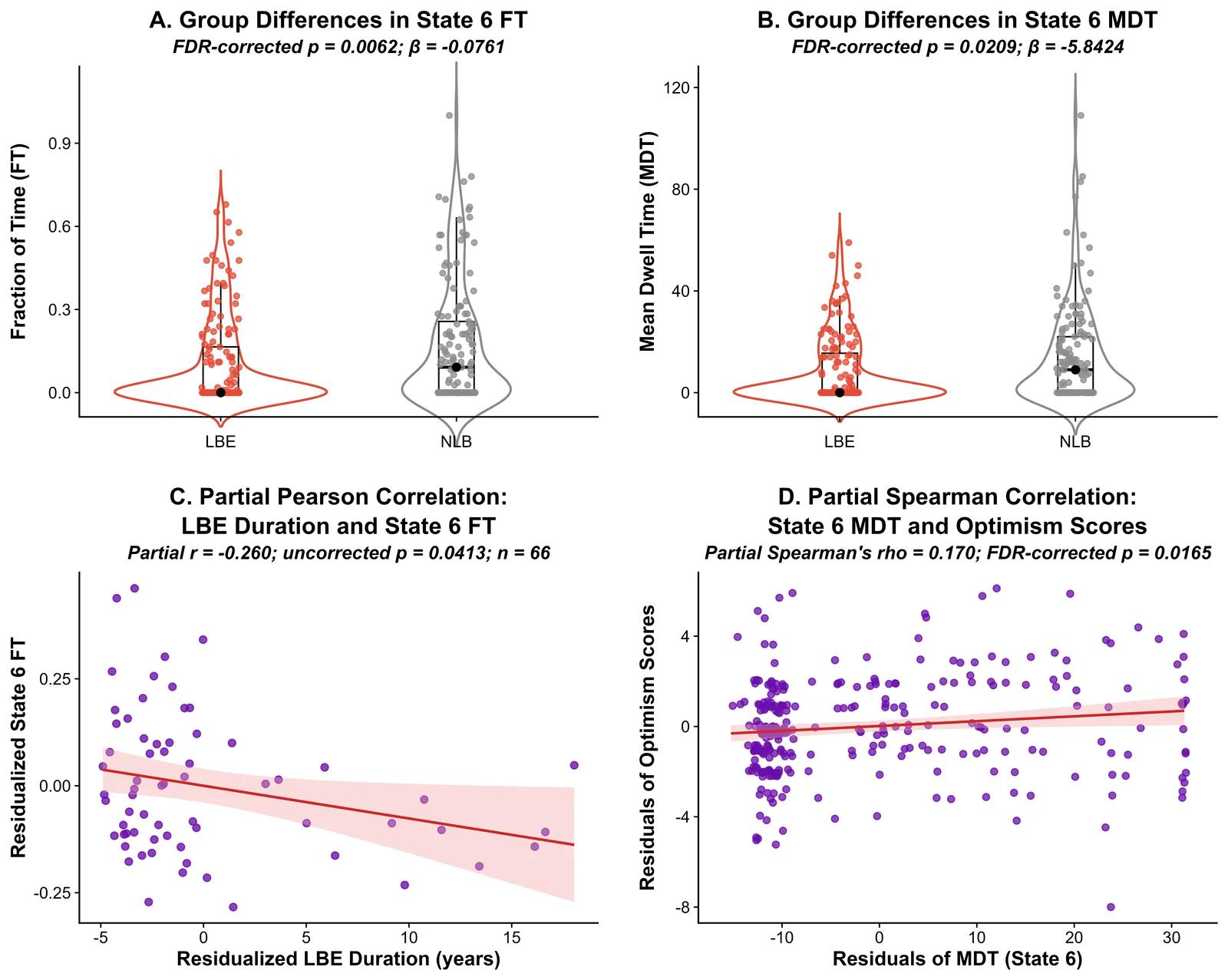
Group differences in dynamic functional connectivity temporal properties and their associations with LBE duration and optimism scores. **(A)** Fraction of time (FT) in State 6 differed significantly between groups, with the LBE group showing reduced FT compared with the NLB group (FDR-corrected p = 0.0062). **(B)** Mean dwell time (MDT) in State 6 was also significantly lower in the LBE group than in the NLB group (FDR-corrected p = 0.0209). **(C)** Among the 66 LBE participants who exhibited State 6, a partial Pearson correlation analysis revealed that longer LBE duration was significantly associated with lower FT in State 6 after controlling for age, sex, years of education, and mean framewise displacement (FD) (r = −0.260, p = 0.0413). **(D)** A partial Spearman correlation analysis revealed a significant positive association between MDT in State 6 and optimism scores after controlling for age, sex, years of education, and mean framewise displacement (FD) (ρ = 0.170, FDR-corrected p = 0.0165). Data in **(A)** and **(B)** are presented as violin plots with overlaid individual data points and boxplots. The fitted regression lines and shaded 95% confidence intervals in **(C)** and **(D)** are shown for visualization purposes only.

As shown in **Figures 4A, B**, significant group differences were observed exclusively in State 6. Compared with the NLB group, the LBE group exhibited significantly reduced FT in State 6 (β = −0.076, 95% CI [−0.121, −0.031], t = −3.317, partial R² = 0.036, FDR-corrected p = 0.0062). MDT in State 6 was also significantly lower in the LBE group (β = −5.842, 95% CI [−9.746, −1.939], t = −2.946, partial R² = 0.029, FDR-corrected p = 0.0209). These effects remained significant after controlling for age, sex, education, and mean FD. Complete results for FT and MDT across all six states and for NT are provided in **Supplementary Table 13**. Sensitivity analyses without education as a covariate yielded similar results (FT: p = 0.0060, FDR-corrected p = 0.0060; MDT: p = 0.0086, FDR-corrected p = 0.0086), indicating that the observed group differences were not solely driven by educational differences. In the outlier sensitivity analyses, the State 6 group differences remained significant after excluding metric-specific outliers for both FT (β = −0.0799, t = −4.414, FDR-corrected p = 8.78 × 10^-5^) and MDT (β = −4.077, t = −2.775, FDR-corrected p = 0.0353; **Supplementary Table 14**). No significant between-group difference was observed in the number of transitions (NT). In the 20-TR sensitivity analysis, the original 30-TR State 6 corresponded closely to 20-TR State 3 (r = 0.998), and the reductions in FT and MDT in the LBE group were reproduced (FDR-corrected p = 0.0006 and 0.0070, respectively; **Supplementary Figures 6, S7**; **Supplementary Tables 7**, **S9**).

### 3.6 Partial correlation analyses

Given that State 6 showed significant group differences in both FT and MDT, we further examined whether these temporal properties were associated with LBE duration and questionnaire-derived measures. Among the 66 LBE participants who exhibited State 6, longer LBE duration was significantly associated with lower FT in State 6 after controlling for age, sex, years of education, and mean FD (partial Pearson’s r = −0.260, p = 0.0413; **Figure 4C**). The association between LBE duration and MDT was not significant (partial Pearson’s r = −0.165, p = 0.200; **Supplementary Table 12**). As shown in **Figure 4D**, MDT in State 6 was positively associated with optimism scores after controlling for the same covariates (partial Spearman’s ρ = 0.170, raw p = 0.0033, FDR-corrected p = 0.0165). For the questionnaire correlation analyses, FDR correction was applied separately across the five questionnaire-derived measures for each State 6 temporal metric (FT and MDT). Complete results are presented in **Supplementary Table 11**.

## 4 Discussion

This study examined dFNC differences in adults with rural left-behind experience (LBE) using an ICA-based dFNC framework. The principal finding was that individuals with LBE showed reduced FT and MDT in State 6, whereas no significant group differences were observed in the temporal properties of the remaining states, the overall number of transitions, sFNC, or state-wise connectivity strength after FDR correction. Thus, within the present dataset and analytical framework, LBE-related differences were detectable in the occurrence and persistence of one specific functional connectivity state, whereas corresponding differences were not detected in time-averaged or state-wise connectivity strength. This pattern was consistent with the conceptual framework of dFNC, in which temporal properties may capture aspects of network organization that are not apparent in time-averaged connectivity measures (25–27). However, it should not be interpreted as evidence that LBE is generally associated with dynamic rather than static connectivity differences; instead, it represents a state-specific temporal finding that requires replication.

A key observation in the present study was that State 6 showed the principal temporal group differences. Although no between-group differences in state-wise connectivity strength survived FDR correction, FT and MDT in State 6 differed significantly between groups. This dissociation suggested that the group differences were expressed in the occurrence and persistence of State 6 rather than in its connectivity strength. Quantitative network-level analyses showed that State 6 was characterized by relatively strong coupling among selected auditory, dorsal attention, frontoparietal, somatomotor, and visual network pairs, together with comparatively weak default mode network involvement. Accordingly, State 6 represented a specific connectivity configuration involving selected sensory and control-related networks, rather than a globally integrated or globally weakly connected state. The coordinated involvement of auditory, visual, somatomotor, attentional, and frontoparietal systems may be broadly consistent with an externally oriented or sensorimotor-related connectivity configuration (14). However, because the present data were acquired during rest, the cognitive or behavioral functions of State 6 cannot be determined directly, and this state should not be considered equivalent to previously reported connectivity states.

Within this framework, the reduced FT and MDT observed in the LBE group indicated less frequent and less sustained expression of this specific connectivity configuration. These group differences remained significant after controlling for age, sex, education, and head motion, although their effect sizes were modest (partial R² = 0.036 for FT and 0.029 for MDT). The reductions in FT and MDT were also reproduced using the shorter 20-TR window and remained significant after the exclusion of metric-specific outliers, supporting the robustness of the principal temporal findings. The combination of stronger coupling among selected sensory and control-related networks and comparatively weak default mode network involvement was broadly consistent with previous research linking early-life social adversity to differences in large-scale brain systems, particularly frontolimbic, default mode, and attentional networks (1, 2, 27–31). Studies in left-behind children similarly reported altered brain network topology and spontaneous activity in motor, sensory, cognitive, and emotion-related regions (8, 9). The present findings extended these observations by identifying differences in temporal network organization in an adult LBE sample; however, because the previous studies were conducted in children and the present study was cross-sectional, the results do not establish that childhood neural differences persisted into adulthood.

Extending beyond this specific population, converging evidence from broader early-life social adversity research provided broader context for interpreting the present findings. Systematic reviews showed that childhood maltreatment was associated with alterations in large-scale brain networks. These included reduced connectivity between the anterior insula and dorsal anterior cingulate cortex, as well as increased amygdala connectivity involving salience, default mode, and prefrontal regulatory systems (27). Meta-analytic evidence also revealed convergent alterations in amygdala–anterior cingulate and amygdala–hippocampal connectivity across different forms of early adversity, suggesting partially shared neural correlates underlying these experiences (30). Although these findings did not directly correspond to State 6, they indicate that early-life social adversity may be associated with distributed differences across large-scale brain networks.

Our findings were also broadly consistent with prior dFNC studies in psychiatric populations, which demonstrated that clinically relevant differences were often expressed in the temporal properties of connectivity states, particularly in terms of state occupancy and dwell time. For example, schizophrenia was associated with reduced time spent in strongly integrated states (25), while major depressive disorder was linked to increased occupancy of less connected states and reduced persistence of more integrated configurations (32). Similar alterations in the temporal organization of brain states were also reported in bipolar disorder (33). Together, these studies suggested that alterations in state occupancy and persistence may occur across different clinical conditions. The present results extended this broader temporal perspective to a non-clinical population, although the State 6 configuration should not be considered directly equivalent to the integrated or less connected states reported in psychiatric disorders. Unlike these psychiatric conditions, LBE represented a form of early-life social adversity in a non-clinical sample. Accordingly, differences in state occupancy and persistence may represent a vulnerability-related neural feature through which early-life social adversity is associated with increased risk for adverse mental health outcomes, although the present cross-sectional findings cannot establish such a pathway.

A central contribution of the present study was the examination of associations of State 6 temporal properties with LBE duration and questionnaire-derived measures. Within the LBE group, longer LBE duration was associated with lower FT in State 6, whereas the association with MDT was not significant. Because FT reflects the proportion of sliding windows assigned to a state, this finding suggested that longer LBE duration was related to lower overall occupancy of State 6 rather than to shorter persistence of individual State 6 episodes. In a related study, Valencia et al. reported that the duration of childhood maltreatment was associated with altered resting-state connectivity between the default mode network and visual and sensorimotor regions (34). The positive association between MDT in State 6 and optimism suggested that longer persistence of this connectivity configuration was related to higher optimism, an aspect of psychological resilience reflecting adaptive capacity, consistent with previous evidence linking psychological resilience to dynamic properties of resting-state brain networks (35). This association was also broadly consistent with prior neuroimaging findings showing that optimism was related to activity and connectivity in medial prefrontal and default mode regions involved in future-oriented cognition and affective regulation (36, 37). In addition, previous studies linked dynamic connectivity patterns to psychological traits such as mindfulness and well-being (38–40). Together, these findings suggested that different temporal properties of State 6 were associated with aspects of early-life experience and adaptive capacity: FT was related to LBE duration, whereas MDT was related to optimism. However, these cross-sectional associations did not establish causal relationships or mediating neural mechanisms.

Another notable aspect of the present results was the dissociation between significant temporal differences and nonsignificant static functional network connectivity findings. Rather than being contradictory, this pattern suggested that sFNC and dFNC may capture complementary aspects of brain network organization. sFNC reflects time-averaged relationships, whereas dFNC captures the temporal variability and recurrence of connectivity states (41). This perspective was consistent with prior findings showing reduced temporal flexibility and meta-state dynamism in adolescents born very preterm (42). In the context of LBE, the present results indicated only that detectable group differences were more evident in the temporal properties of network states under the current analytical framework; they should not be interpreted as evidence for a general absence of static connectivity differences.

Several limitations should be noted. First, the resting-state acquisition was relatively short (160 volumes; approximately 5 min and 20 s), with 150 volumes retained after removal of the first 10 volumes. The limited amount of within-subject data may have reduced the reliability of dFNC estimates, particularly individual-level temporal metrics. Although the principal FT and MDT findings were reproduced using a 20-TR window, this sensitivity analysis did not fully overcome the limitation of the short acquisition. Second, dFNC estimation depends on methodological choices such as window length and clustering strategy. Although complementary cluster-validity indices, split-half reproducibility, and the shorter-window sensitivity analysis supported the principal findings, the gap statistic favored K = 8, indicating some uncertainty regarding the optimal number of states. Third, group classification was based solely on rural left-behind experience, whereas other forms of early-life social adversity, social support, and caregiving quality were not assessed. Therefore, participants in the NLB group may also have experienced other substantial adversities, limiting the extent to which the observed differences can be attributed specifically to LBE. Fourth, the cross-sectional design precludes causal inference. Fifth, the observed effects, while statistically significant, explain a modest proportion of variance, which is typical for individual-difference neuroimaging research (43). Sixth, the relatively narrow age range of 20–40 years and the high educational attainment of the sample may limit the generalizability of the findings to populations with different age and educational backgrounds. Finally, potential interaction effects were not examined in the present study. Future research should investigate whether additional factors, such as current stress levels or social support, may moderate the observed associations (44).

In conclusion, the present findings indicated that rural left-behind experience was associated with state-specific differences in dynamic brain organization. Specifically, reduced fraction of time (FT) and mean dwell time (MDT) in State 6 indicated less frequent occurrence and shorter persistence of this specific connectivity configuration in the LBE group. These group differences remained significant after controlling for age, sex, years of education, and mean FD, remained robust after the exclusion of metric-specific outliers, and were reproduced using the shorter 20-TR window. In addition, longer LBE duration was associated with lower FT in State 6, whereas higher MDT was associated with greater optimism. Together, these findings provided preliminary correlational evidence that LBE was associated with altered temporal engagement with a specific connectivity state, while no significant group differences were detected in sFNC or state-wise connectivity strength under the current analytical framework.

## Data Availability

The raw data supporting the conclusions of this article will be made available by the authors, without undue reservation.
