# Supplementary material for: Altered dynamic functional connectivity in adults with rural left-behind experience: a resting-state fMRI study
Source: Front Psychiatry. 2026 Aug 26;17:1900163. doi: 10.3389/fpsyt.2026.1900163 (PMC13553950; doi:10.3389/fpsyt.2026.1900163)
Supplement: Supplementary file 1 [file SupplementaryFile1.docx]

Supplementary Material

**Altered Dynamic Functional Connectivity in Adults With Rural Left-Behind Experience: A Resting-State fMRI Study**

# 1 Supplementary Data

## 1.1 Supplementary Methods

**Validation of the number of dFNC states**

Candidate clustering solutions from K = 2 to K = 10 were evaluated using four complementary criteria: the elbow criterion based on the ratio of within-cluster to between-cluster dispersion, the mean silhouette coefficient, the gap statistic, and repeated split-half reproducibility. For the split-half analysis, participants were randomly divided into two approximately equal subsamples. Independent k-means clustering was performed in each half, the resulting centroids were matched using an optimal one-to-one assignment based on centroid correlations, and agreement was summarized using the adjusted Rand index (ARI). The random split procedure was repeated 100 times. The six-state solution was retained based on convergence across complementary indices and the parsimony of the solution.

**Within-state consistency, cross-subject reproducibility, and between-state separation**

Within-state consistency was quantified by calculating the Pearson correlation between each vectorized sliding-window dFNC pattern and the centroid of the state to which that window was assigned. For each state, the number of windows, mean, standard deviation, median, interquartile range, and 95% confidence interval of the window-to-centroid correlations were summarized.

Cross-subject reproducibility was evaluated by averaging all windows assigned to a given state within each participant and correlating the participant-specific mean pattern with the corresponding group-level centroid. Participants contributing at least three windows to a state were included. Pairwise correlations among participant-specific state patterns were additionally summarized. Between-state separation was quantified using pairwise Pearson correlations and Euclidean distances among the six group-level centroids.

**Network-level characterization of State 6**

The nine independent components included in the dFNC analysis were assigned to six canonical networks: auditory network (AN), default mode network (DMN), dorsal attention network (DAN), frontoparietal network (FPN), somatomotor network (SMN), and visual network (VN). Mean Fisher z-transformed connectivity values were calculated for 18 within- and between-network pairs. To quantify the distinctiveness of State 6, participant-level network-pair values in State 6 were compared with the mean of the corresponding values across the other five states using paired tests, followed by Benjamini-Hochberg false discovery rate (FDR) correction across the 18 network pairs.

**Assessment of unequal group contributions to the pooled State 6 centroid**

The number of State 6 windows contributed by the LBE and NLB groups was counted under the original pooled 30-TR clustering solution. Group-specific State 6 centroids were reconstructed by averaging the windows assigned to State 6 separately within each group. Pearson correlations were calculated between the two group-specific centroids, between each group-specific centroid and the original pooled centroid, and between an equal-group-weighted centroid and the original pooled centroid.

**Window-length sensitivity analysis**

Considering the potential influence of window length on dFNC estimation, the complete analytical pipeline was repeated using a shorter 20-TR (40-s) window, with all other analytical settings held constant, including the ICA decomposition, selected components, step size, number of clusters (K = 6), group definitions, and statistical covariates. The 20-TR state centroids were matched to the original 30-TR centroids using an optimal one-to-one assignment that maximized the summed Pearson correlation between vectorized centroid patterns.

For the matched state corresponding to the original 30-TR State 6, group differences in fraction of time (FT) and mean dwell time (MDT) were tested using linear models controlling for mean framewise displacement, age, sex, and education. FDR correction was applied across the six states within each temporal metric. Partial Spearman correlations between the matched-state MDT and five questionnaire-derived measures were calculated after residualizing both variables for the same covariates.

**Complete statistical reporting and outlier sensitivity analysis**

To provide complete reporting, the original covariate-adjusted linear model was rerun for fraction of time (FT) and mean dwell time (MDT) in all six states and for the overall number of transitions (NT). The same model was also applied to all 36 static functional network connectivity (sFNC) edges and to all 36 state-specific dFNC edges within each of the six states. All models controlled for mean framewise displacement, age, sex, and years of education, and the adjusted group coefficient represented LBE minus NLB. State-specific dFNC edge analyses included participants with an available subject-level connectivity pattern for the corresponding state. Benjamini-Hochberg FDR correction was applied across the six states separately for FT and MDT, across the 36 sFNC edges, and separately across the 36 dFNC edges within each state.

For the outlier sensitivity analysis, State 6 FT and MDT outliers were identified separately within the LBE and NLB groups using the 1.5-interquartile-range criterion. For each metric, participants with outlying values were excluded and the original covariate-adjusted linear model was repeated. FDR correction was recalculated across the six states within the corresponding temporal metric in the reduced sample.

## 1.2 Supplementary Results

**Validation of the six-state solution**

The elbow curve showed a marked change in slope at K = 6. The mean silhouette coefficient and split-half ARI also reached their maxima at K = 6, whereas the gap statistic reached its maximum at K = 8. Because the elbow, silhouette, and split-half reproducibility criteria converged on K = 6, and because the six-state solution was more parsimonious than the eight-state alternative, K = 6 was retained (Supplementary Figure S1).

**State consistency, reproducibility, and separation**

Across the six states, mean window-to-centroid correlations ranged from 0.468 to 0.762. State 2 showed the highest within-state consistency (mean r = 0.762, SD = 0.085), whereas State 3 showed the lowest consistency and greatest variability (mean r = 0.468, SD = 0.142). State 6 showed moderate-to-high internal consistency (mean r = 0.661, SD = 0.117; median = 0.677, interquartile range = 0.595-0.744; Supplementary Figure S2 and Supplementary Table S1).

Mean subject-to-centroid correlations ranged from 0.550 to 0.793 across states. For State 6, the mean subject-to-centroid correlation was 0.700 (SD = 0.113), and the median between-subject correlation was 0.500, indicating moderate cross-subject reproducibility (Supplementary Figure S4 and Supplementary Table S2). Pairwise centroid correlations ranged from 0.307 to 0.761, and Euclidean distances ranged from 1.425 to 2.811. State 6 was most similar to State 3 (r = 0.752; Euclidean distance = 1.488), but remained non-identical to all other states (Supplementary Figure S3 and Supplementary Tables S3-S4).

**Quantitative network-level profile of State 6**

In the State 6 centroid, the largest positive network-pair values were observed for AN-SMN (z = 0.595), FPN-FPN (z = 0.504), SMN-VN (z = 0.439), and AN-VN (z = 0.433). Connectivity involving the DMN was comparatively weak, including negative AN-DMN, DMN-DAN, and DMN-DMN values. Compared with the mean of the other five states, State 6 showed significantly stronger positive coupling for AN-FPN, AN-VN, DMN–SMN, DAN-FPN, DAN-SMN, DAN-VN, FPN-SMN, FPN-VN, and SMN-VN after FDR correction (Supplementary Figure S5 and Supplementary Table S8).

**Influence of unequal group contributions**

The LBE group contributed 1,725 State 6 windows (38.9%), whereas the NLB group contributed 2,706 windows (61.1%). Despite this difference, the LBE- and NLB-specific State 6 centroids were highly correlated (r = 0.986). The LBE- and NLB-specific centroids were also strongly correlated with the pooled centroid (r = 0.995 and r = 0.998, respectively), and the equal-group-weighted centroid was nearly identical to the pooled centroid (r = 0.9998). These findings indicate that unequal window counts did not materially alter the topology of the pooled State 6 centroid (Supplementary Table S6).

**Window-length sensitivity analysis**

All six 20-TR state centroids showed very high correspondence with the original 30-TR states. Matched Pearson correlations ranged from 0.993 to 0.999, with a mean matched correlation of 0.996. The original 30-TR State 6 corresponded to 20-TR State 3 (r = 0.998; Supplementary Figure S6 and Supplementary Table S7).

The principal temporal group differences were reproduced in the matched 20-TR State 3. The LBE group showed a lower FT than the NLB group (LBE mean = 0.110, SD = 0.136; NLB mean = 0.178, SD = 0.185; adjusted LBE-minus-NLB coefficient = -0.0757; t = -3.955; raw p = 0.000096; FDR-corrected p = 0.000576). The LBE group also showed a lower MDT (LBE mean = 8.226, SD = 9.729; NLB mean = 12.868, SD = 15.128; adjusted LBE-minus-NLB coefficient = -4.943; t = -3.277; raw p = 0.001175; FDR-corrected p = 0.007048; Supplementary Figure S7A-B and Supplementary Table S9).

The partial Spearman association between MDT in the matched 20-TR State 3 and optimism was positive and nominally significant (ρ = 0.128, uncorrected p = 0.0277; Supplementary Figure S7C and Supplementary Table S10).

**Complete statistical reporting and outlier sensitivity**

Complete temporal results are provided in Supplementary Table S13. State 6 remained the only state showing significant group differences after FDR correction (FT: adjusted beta = -0.0761, t = -3.317, FDR-corrected p = 0.0062; MDT: adjusted beta = -5.842, t = -2.946, FDR-corrected p = 0.0209). No sFNC edge survived FDR correction, and no state-specific dFNC edge survived FDR correction (Supplementary Tables S15-S16).

Using the group-specific 1.5-interquartile-range criterion, 12 LBE and 7 NLB participants were identified as outliers for State 6 FT, whereas 6 LBE and 7 NLB participants were identified as outliers for State 6 MDT. After exclusion, the State 6 FT group difference remained significant (adjusted beta = -0.0799, t = -4.414, FDR-corrected p = 8.78 × 10⁻⁵), and the State 6 MDT group difference also remained significant (adjusted beta = -4.077, t = -2.775, FDR-corrected p = 0.0353; Supplementary Table S14). These sensitivity analyses indicated that the principal temporal findings were not attributable to a small number of extreme observations.

# 2 Supplementary Figures and Tables

## 2.1 Supplementary Tables

**Supplementary Table S1.** Window-to-centroid consistency for the six dFNC states.

| **State** | **Windows (n)** | **Mean r** | **SD** | **Median r** | **IQR** | **95% CI** |
| --- | --- | --- | --- | --- | --- | --- |
| 1 | 5700 | 0.701 | 0.107 | 0.707 | 0.632-0.784 | 0.698-0.704 |
| 2 | 3746 | 0.762 | 0.085 | 0.769 | 0.703-0.828 | 0.759-0.764 |
| 3 | 9651 | 0.468 | 0.142 | 0.473 | 0.368-0.569 | 0.465-0.471 |
| 4 | 3634 | 0.690 | 0.103 | 0.695 | 0.629-0.763 | 0.686-0.693 |
| 5 | 5211 | 0.708 | 0.095 | 0.710 | 0.644-0.777 | 0.705-0.710 |
| 6 | 4431 | 0.661 | 0.117 | 0.677 | 0.595-0.744 | 0.657-0.664 |

*Note.* r values are Pearson correlations between each sliding-window dFNC vector and its assigned state centroid. IQR, interquartile range; CI, confidence interval.

**Supplementary Table S2.** Cross-subject reproducibility of the six dFNC states.

| **State** | **Subjects (n)** | **Median windows** | **Mean subject-to-centroid r** | **SD** | **Median between-subject r** | **Between-subject IQR** |
| --- | --- | --- | --- | --- | --- | --- |
| 1 | 167 | 27 | 0.734 | 0.107 | 0.548 | 0.439-0.651 |
| 2 | 94 | 37 | 0.793 | 0.086 | 0.639 | 0.551-0.724 |
| 3 | 244 | 36 | 0.550 | 0.136 | 0.304 | 0.184-0.424 |
| 4 | 126 | 23.5 | 0.726 | 0.091 | 0.538 | 0.437-0.633 |
| 5 | 160 | 28 | 0.743 | 0.098 | 0.564 | 0.461-0.655 |
| 6 | 143 | 24.5 | 0.700 | 0.113 | 0.500 | 0.387-0.604 |

*Note.* Participant-specific mean state patterns were included when a participant contributed at least three windows to the corresponding state.

**Supplementary Table S3.** Pairwise Pearson correlations among the six group-level state centroids.

| **State** | **State1** | **State2** | **State3** | **State4** | **State5** | **State6** |
| --- | --- | --- | --- | --- | --- | --- |
| State1 | 1.000 | 0.307 | 0.702 | 0.502 | 0.746 | 0.607 |
| State2 | 0.307 | 1.000 | 0.715 | 0.607 | 0.486 | 0.690 |
| State3 | 0.702 | 0.715 | 1.000 | 0.712 | 0.761 | 0.752 |
| State4 | 0.502 | 0.607 | 0.712 | 1.000 | 0.628 | 0.675 |
| State5 | 0.746 | 0.486 | 0.761 | 0.628 | 1.000 | 0.558 |
| State6 | 0.607 | 0.690 | 0.752 | 0.675 | 0.558 | 1.000 |

*Note.* The diagonal equals 1.000 by definition.

**Supplementary Table S4.** Pairwise Euclidean distances among the six group-level state centroids.

| **State** | **State1** | **State2** | **State3** | **State4** | **State5** | **State6** |
| --- | --- | --- | --- | --- | --- | --- |
| State1 | 0.000 | 2.811 | 1.482 | 2.086 | 1.497 | 1.849 |
| State2 | 2.811 | 0.000 | 2.015 | 2.188 | 2.452 | 2.109 |
| State3 | 1.482 | 2.015 | 0.000 | 1.568 | 1.425 | 1.488 |
| State4 | 2.086 | 2.188 | 1.568 | 0.000 | 1.849 | 1.763 |
| State5 | 1.497 | 2.452 | 1.425 | 1.849 | 0.000 | 2.101 |
| State6 | 1.849 | 2.109 | 1.488 | 1.763 | 2.101 | 0.000 |

*Note.* Larger values indicate greater separation between state centroids.

**Supplementary Table S5.** Independent-component order and network assignment used in the dFNC analysis.

| **FNC position** | **IC number** | **Network** |
| --- | --- | --- |
| 1 | 6 | AN |
| 2 | 4 | DMN |
| 3 | 20 | DMN |
| 4 | 5 | DAN |
| 5 | 13 | DAN |
| 6 | 11 | FPN |
| 7 | 12 | FPN |
| 8 | 7 | SMN |
| 9 | 10 | VN |

*Note.* AN, auditory network; DMN, default mode network; DAN, dorsal attention network; FPN, frontoparietal network; SMN, somatomotor network; VN, visual network.

**Supplementary Table S6.** Assessment of unequal LBE and NLB contributions to the pooled State 6 centroid.

| **Quantity** | **Result** |
| --- | --- |
| LBE State 6 windows | 1,725 (38.9%) |
| NLB State 6 windows | 2,706 (61.1%) |
| LBE-specific vs NLB-specific centroid | r = 0.986 |
| LBE-specific vs pooled centroid | r = 0.995 |
| NLB-specific vs pooled centroid | r = 0.998 |
| Equal-group-weighted vs pooled centroid | r = 0.9998 |

**Supplementary Table S7.** Optimal one-to-one matching between the original 30-TR and sensitivity-analysis 20-TR state centroids.

| **Original 30-TR state** | **Matched 20-TR state** | **Pearson r** |
| --- | --- | --- |
| 1 | 5 | 0.996 |
| 2 | 2 | 0.999 |
| 3 | 4 | 0.993 |
| 4 | 6 | 0.994 |
| 5 | 1 | 0.996 |
| 6 | 3 | 0.998 |

*Note.* The mean matched centroid correlation was r = 0.996.

**Supplementary Table S8.** Network-pair profile of State 6 and paired comparisons with the mean of the other five states.

| **Network pair** | **n** | **State 6 mean** | **Other states mean** | **Difference** | **Cohen's dz** | **t** | **Raw p** | **FDR p** |
| --- | --- | --- | --- | --- | --- | --- | --- | --- |
| AN-DMN | 143 | -0.185 | -0.253 | 0.068 | 0.323 | 3.860 | 1.72e-04 | 3.09e-04 |
| AN-DAN | 143 | 0.316 | 0.302 | 0.014 | 0.073 | 0.875 | 0.3828 | 0.4053 |
| AN-FPN | 143 | 0.180 | -0.066 | 0.246 | 1.068 | 12.772 | 2.36e-25 | 1.41e-24 |
| AN-SMN | 143 | 0.563 | 0.542 | 0.021 | 0.078 | 0.937 | 0.3505 | 0.3943 |
| AN-VN | 143 | 0.384 | 0.238 | 0.146 | 0.551 | 6.583 | 8.27e-10 | 2.48e-09 |
| DMN-DMN | 143 | -0.022 | 0.127 | -0.149 | -0.500 | -5.978 | 1.74e-08 | 3.90e-08 |
| DMN-DAN | 143 | -0.077 | -0.166 | 0.090 | 0.509 | 6.081 | 1.05e-08 | 2.69e-08 |
| DMN-FPN | 143 | 0.123 | 0.120 | 0.002 | 0.013 | 0.152 | 0.8790 | 0.8790 |
| DMN-SMN | 143 | 0.104 | 0.044 | 0.060 | 0.278 | 3.330 | 0.0011 | 0.0018 |
| DMN-VN | 143 | 0.103 | 0.126 | -0.023 | -0.092 | -1.098 | 0.2739 | 0.3287 |
| DAN-DAN | 143 | 0.103 | 0.146 | -0.043 | -0.127 | -1.520 | 0.1307 | 0.1681 |
| DAN-FPN | 143 | 0.211 | 0.064 | 0.146 | 0.938 | 11.217 | 2.64e-21 | 1.19e-20 |
| DAN-SMN | 143 | 0.310 | 0.238 | 0.071 | 0.360 | 4.302 | 3.14e-05 | 6.27e-05 |
| DAN-VN | 143 | 0.232 | 0.066 | 0.166 | 0.636 | 7.606 | 3.54e-12 | 1.28e-11 |
| FPN-FPN | 143 | 0.489 | 0.447 | 0.042 | 0.146 | 1.747 | 0.0828 | 0.1146 |
| FPN-SMN | 143 | 0.243 | -0.019 | 0.263 | 1.222 | 14.614 | 4.26e-30 | 3.83e-29 |
| FPN-VN | 143 | 0.197 | -0.072 | 0.269 | 1.260 | 15.071 | 2.95e-31 | 5.32e-30 |
| SMN-VN | 143 | 0.399 | 0.312 | 0.087 | 0.272 | 3.248 | 0.0014 | 0.0022 |

*Note.* Positive differences indicate higher connectivity in State 6. P values were FDR-corrected across the 18 network pairs. Network abbreviations are defined in Supplementary Table S5.

**Supplementary Table S9.** Group differences in temporal properties of the matched 20-TR State 3.

| **Metric** | **LBE mean** | **LBE SD** | **NLB mean** | **NLB SD** | **Adjusted beta (LBE-NLB)** | **t** | **Raw p** | **FDR p** |
| --- | --- | --- | --- | --- | --- | --- | --- | --- |
| FT | 0.110 | 0.136 | 0.178 | 0.185 | -0.0757 | -3.955 | 0.000096 | 0.000576 |
| MDT | 8.226 | 9.729 | 12.868 | 15.128 | -4.943 | -3.277 | 0.001175 | 0.007048 |

*Note.* Linear models controlled for mean framewise displacement, age, sex, and education. FDR correction was applied across the six states separately for FT and MDT.

**Supplementary Table S10.** Partial Spearman correlations between MDT in the matched 20-TR State 3 and questionnaire-derived measures.

| **Questionnaire-derived measure** | **Partial Spearman ρ** | **p** |
| --- | --- | --- |
| SDS score | -0.076 | 0.1905 |
| CD-RISC score | 0.095 | 0.1013 |
| Optimism score | 0.128 | 0.0277 |
| Strength score | 0.082 | 0.1575 |
| Tenacity score | 0.069 | 0.2373 |

*Note.* Both MDT and each questionnaire-derived measure were residualized for mean framewise displacement, age, sex, and education.

**Supplementary Table S11.** Partial Spearman correlations of State 6 FT and MDT with questionnaire-derived measures in the primary 30-TR analysis.

| **Panel A. Fraction of time (FT)** | | | | |
| --- | --- | --- | --- | --- |
| **Questionnaire-derived measure** | **Partial Spearman ρ** | **Raw p** | **FDR-corrected p** | **n** |
| SDS score | -0.062 | 0.2896 | 0.2896 | 297 |
| CD-RISC score | 0.112 | 0.0535 | 0.1128 | 297 |
| Optimism score | 0.148 | 0.0105 | 0.0523 | 297 |
| Strength score | 0.106 | 0.0677 | 0.1128 | 297 |
| Tenacity score | 0.092 | 0.1121 | 0.1401 | 297 |
| **Panel B. Mean dwell time (MDT)** | | | | |
| **Questionnaire-derived measure** | **Partial Spearman ρ** | **Raw p** | **FDR-corrected p** | **n** |
| SDS score | -0.081 | 0.1638 | 0.1638 | 297 |
| CD-RISC score | 0.131 | 0.0238 | 0.0594 | 297 |
| Optimism score | 0.170 | 0.0033 | 0.0165 | 297 |
| Strength score | 0.109 | 0.0603 | 0.0753 | 297 |
| Tenacity score | 0.119 | 0.0407 | 0.0679 | 297 |

*Note.* Partial Spearman correlations controlled for age, sex, years of education, and mean framewise displacement. FDR correction was applied separately across the five questionnaire-derived measures for each State 6 temporal metric (FT and MDT).

**Supplementary Table S12.** Partial Pearson correlations of LBE duration with State 6 temporal properties in the primary 30-TR analysis.

| **Temporal metric** | **n** | **Partial Pearson r** | **p** |
| --- | --- | --- | --- |
| FT | 66 | -0.260 | 0.0413 |
| MDT | 66 | -0.165 | 0.2000 |

*Note.* Analyses were restricted to LBE participants who exhibited State 6 and controlled for age, sex, years of education, and mean framewise displacement. Uncorrected two-sided p values are reported.

**Supplementary Table S13.** Complete group comparisons of primary 30-TR dFNC temporal properties.

| **Metric** | **State** | **LBE mean (SD)** | **NLB mean (SD)** | **Adjusted beta** | **95% CI** | **t** | **Raw p** | **FDR p** | **Partial R2** |
| --- | --- | --- | --- | --- | --- | --- | --- | --- | --- |
| FT | 1 | 0.182 (0.248) | 0.170 (0.225) | 0.0145 | -0.0412 to 0.0703 | 0.513 | 0.6081 | 0.7200 | 0.001 |
| FT | 2 | 0.089 (0.191) | 0.144 (0.246) | -0.0514 | -0.1027 to -0.0002 | -1.975 | 0.0493 | 0.0843 | 0.013 |
| FT | 3 | 0.324 (0.249) | 0.271 (0.235) | 0.0614 | 0.0047 to 0.1182 | 2.133 | 0.0338 | 0.0843 | 0.015 |
| FT | 4 | 0.135 (0.209) | 0.088 (0.163) | 0.0426 | -0.0011 to 0.0863 | 1.917 | 0.0562 | 0.0843 | 0.012 |
| FT | 5 | 0.166 (0.215) | 0.155 (0.203) | 0.0090 | -0.0402 to 0.0581 | 0.359 | 0.7200 | 0.7200 | 0.000 |
| FT | 6 | 0.104 (0.165) | 0.171 (0.219) | -0.0761 | -0.1213 to -0.0309 | -3.317 | 0.0010 | 0.0062 | 0.036 |
| MDT | 1 | 15.962 (22.282) | 13.338 (18.329) | 3.245 | -1.553 to 8.043 | 1.331 | 0.1842 | 0.2211 | 0.006 |
| MDT | 2 | 8.319 (18.750) | 12.469 (21.807) | -3.873 | -8.617 to 0.870 | -1.607 | 0.1091 | 0.1756 | 0.009 |
| MDT | 3 | 22.433 (20.064) | 19.230 (20.481) | 3.798 | -0.957 to 8.553 | 1.572 | 0.1170 | 0.1756 | 0.008 |
| MDT | 4 | 10.778 (16.785) | 7.129 (12.257) | 3.498 | 0.042 to 6.954 | 1.992 | 0.0473 | 0.1419 | 0.013 |
| MDT | 5 | 12.752 (16.712) | 12.034 (16.217) | 0.549 | -3.307 to 4.404 | 0.280 | 0.7797 | 0.7797 | 0.000 |
| MDT | 6 | 8.800 (13.498) | 14.202 (19.406) | -5.842 | -9.746 to -1.939 | -2.946 | 0.0035 | 0.0209 | 0.029 |
| NT | Overall | 3.625 (1.771) | 3.917 (1.734) | -0.254 | -0.665 to 0.157 | -1.217 | 0.2244 | NA | 0.005 |

*Note.* Linear models controlled for mean framewise displacement, age, sex, and years of education. Adjusted beta values represent LBE minus NLB. FDR correction was applied separately across the six states for FT and MDT. NT was an overall measure and was not FDR-corrected.

**Supplementary Table S14.** Outlier sensitivity analyses for State 6 FT and MDT.

| **Metric** | **Analysis** | **Excluded LBE/NLB** | **N** | **Adjusted beta** | **95% CI** | **t** | **Raw p** | **FDR p** | **Partial R2** |
| --- | --- | --- | --- | --- | --- | --- | --- | --- | --- |
| FT | Full sample | 0/0 | 297 | -0.0761 | -0.1213 to -0.0309 | -3.317 | 0.0010 | 0.0062 | 0.036 |
| FT | Outliers excluded | 12/7 | 278 | -0.0799 | -0.1156 to -0.0443 | -4.414 | 1.46e-05 | 8.78 × 10⁻⁵ | 0.067 |
| MDT | Full sample | 0/0 | 297 | -5.842 | -9.746 to -1.939 | -2.946 | 0.0035 | 0.0209 | 0.029 |
| MDT | Outliers excluded | 6/7 | 284 | -4.077 | -6.969 to -1.185 | -2.775 | 0.0059 | 0.0353 | 0.027 |

*Note.* Outliers were identified separately within each group as values below Q1 - 1.5 x IQR or above Q3 + 1.5 x IQR. Models controlled for mean framewise displacement, age, sex, and years of education. FDR correction was recalculated across the six states within each temporal metric after the corresponding exclusions.

**Supplementary Table S15.** Complete covariate-adjusted edge-level sFNC group-comparison results.

| **Edge** | **Component/network pair** | **LBE mean** | **NLB mean** | **Adjusted beta** | **t** | **Raw p** | **FDR p** | **Partial R2** |
| --- | --- | --- | --- | --- | --- | --- | --- | --- |
| 1 | IC6 (AN)-IC4 (DMN) | -0.009 | 0.006 | -0.023 | -0.788 | 0.4316 | 0.8286 | 0.002 |
| 2 | IC6 (AN)-IC20 (DMN) | -0.524 | -0.553 | 0.033 | 1.185 | 0.2370 | 0.6877 | 0.005 |
| 3 | IC6 (AN)-IC5 (DAN) | 0.132 | 0.066 | 0.058 | 1.680 | 0.0941 | 0.4379 | 0.010 |
| 4 | IC6 (AN)-IC13 (DAN) | 0.493 | 0.518 | -0.036 | -1.194 | 0.2336 | 0.6877 | 0.005 |
| 5 | IC6 (AN)-IC11 (FPN) | -0.072 | -0.052 | -0.020 | -0.732 | 0.4646 | 0.8286 | 0.002 |
| 6 | IC6 (AN)-IC12 (FPN) | -0.063 | -0.060 | -0.013 | -0.483 | 0.6292 | 0.9061 | 0.001 |
| 7 | IC6 (AN)-IC7 (SMN) | 0.522 | 0.484 | 0.023 | 0.758 | 0.4489 | 0.8286 | 0.002 |
| 8 | IC6 (AN)-IC10 (VN) | 0.141 | 0.195 | -0.050 | -1.574 | 0.1166 | 0.4379 | 0.008 |
| 9 | IC4 (DMN)-IC20 (DMN) | 0.105 | 0.132 | -0.028 | -0.919 | 0.3589 | 0.8286 | 0.003 |
| 10 | IC4 (DMN)-IC5 (DAN) | 0.162 | 0.156 | 0.011 | 0.376 | 0.7071 | 0.9151 | 0.000 |
| 11 | IC4 (DMN)-IC13 (DAN) | 0.053 | 0.059 | -0.010 | -0.311 | 0.7560 | 0.9234 | 0.000 |
| 12 | IC4 (DMN)-IC11 (FPN) | -0.018 | 0.049 | -0.072 | -2.537 | 0.0117 | 0.2108 | 0.022 |
| 13 | IC4 (DMN)-IC12 (FPN) | 0.300 | 0.323 | -0.019 | -0.630 | 0.5294 | 0.8286 | 0.001 |
| 14 | IC4 (DMN)-IC7 (SMN) | 0.215 | 0.222 | -0.000 | -0.010 | 0.9920 | 0.9920 | 0.000 |
| 15 | IC4 (DMN)-IC10 (VN) | 0.294 | 0.276 | 0.012 | 0.378 | 0.7058 | 0.9151 | 0.000 |
| 16 | IC20 (DMN)-IC5 (DAN) | -0.372 | -0.315 | -0.054 | -1.576 | 0.1161 | 0.4379 | 0.008 |
| 17 | IC20 (DMN)-IC13 (DAN) | -0.548 | -0.551 | 0.003 | 0.101 | 0.9194 | 0.9457 | 0.000 |
| 18 | IC20 (DMN)-IC11 (FPN) | -0.015 | -0.034 | 0.019 | 0.688 | 0.4918 | 0.8286 | 0.002 |
| 19 | IC20 (DMN)-IC12 (FPN) | 0.095 | 0.103 | 0.006 | 0.184 | 0.8543 | 0.9455 | 0.000 |
| 20 | IC20 (DMN)-IC7 (SMN) | -0.102 | -0.173 | 0.075 | 2.544 | 0.0115 | 0.2108 | 0.022 |
| 21 | IC20 (DMN)-IC10 (VN) | 0.008 | 0.007 | -0.005 | -0.135 | 0.8930 | 0.9455 | 0.000 |
| 22 | IC5 (DAN)-IC13 (DAN) | 0.224 | 0.116 | 0.109 | 2.306 | 0.0218 | 0.2621 | 0.018 |
| 23 | IC5 (DAN)-IC11 (FPN) | 0.042 | 0.054 | -0.008 | -0.293 | 0.7695 | 0.9234 | 0.000 |
| 24 | IC5 (DAN)-IC12 (FPN) | 0.140 | 0.134 | 0.012 | 0.370 | 0.7118 | 0.9151 | 0.000 |
| 25 | IC5 (DAN)-IC7 (SMN) | -0.040 | -0.033 | 0.006 | 0.155 | 0.8769 | 0.9455 | 0.000 |
| 26 | IC5 (DAN)-IC10 (VN) | -0.217 | -0.278 | 0.062 | 1.630 | 0.1041 | 0.4379 | 0.009 |
| 27 | IC13 (DAN)-IC11 (FPN) | 0.113 | 0.142 | -0.038 | -1.157 | 0.2483 | 0.6877 | 0.005 |
| 28 | IC13 (DAN)-IC12 (FPN) | -0.073 | -0.065 | -0.026 | -0.837 | 0.4036 | 0.8286 | 0.002 |
| 29 | IC13 (DAN)-IC7 (SMN) | 0.433 | 0.465 | -0.039 | -1.027 | 0.3051 | 0.7846 | 0.004 |
| 30 | IC13 (DAN)-IC10 (VN) | 0.334 | 0.361 | -0.026 | -0.529 | 0.5973 | 0.8960 | 0.001 |
| 31 | IC11 (FPN)-IC12 (FPN) | 0.408 | 0.426 | -0.017 | -0.634 | 0.5263 | 0.8286 | 0.001 |
| 32 | IC11 (FPN)-IC7 (SMN) | -0.060 | -0.010 | -0.060 | -2.173 | 0.0306 | 0.2753 | 0.016 |
| 33 | IC11 (FPN)-IC10 (VN) | -0.086 | -0.049 | -0.046 | -1.552 | 0.1216 | 0.4379 | 0.008 |
| 34 | IC12 (FPN)-IC7 (SMN) | -0.003 | 0.004 | -0.020 | -0.747 | 0.4556 | 0.8286 | 0.002 |
| 35 | IC12 (FPN)-IC10 (VN) | -0.075 | -0.029 | -0.055 | -1.754 | 0.0804 | 0.4379 | 0.010 |
| 36 | IC7 (SMN)-IC10 (VN) | 0.247 | 0.238 | 0.010 | 0.250 | 0.8031 | 0.9326 | 0.000 |

*Note.* All 297 participants were included. Unadjusted group means are shown for description; adjusted beta and t values were obtained from linear models controlling for mean framewise displacement, age, sex, and years of education. Adjusted beta values represent LBE minus NLB. FDR correction was applied across the 36 sFNC edges. No edge survived FDR correction. Network abbreviations are defined in Supplementary Table S5.

**Supplementary Table S16.** Complete covariate-adjusted edge-level dFNC group-comparison results across all six states.

| **State** | **Edge** | **Component/network pair** | **N LBE/NLB** | **LBE mean** | **NLB mean** | **Adjusted beta** | **t** | **Raw p** | **FDR p** |
| --- | --- | --- | --- | --- | --- | --- | --- | --- | --- |
| 1 | 1 | IC6 (AN)-IC4 (DMN) | 72/67 | -0.017 | -0.016 | 0.005 | 0.090 | 0.9281 | 0.9954 |
| 1 | 2 | IC6 (AN)-IC20 (DMN) | 72/67 | -0.630 | -0.687 | 0.066 | 1.427 | 0.1560 | 0.9196 |
| 1 | 3 | IC6 (AN)-IC5 (DAN) | 72/67 | 0.413 | 0.457 | -0.035 | -0.632 | 0.5285 | 0.9196 |
| 1 | 4 | IC6 (AN)-IC13 (DAN) | 72/67 | 0.619 | 0.661 | -0.034 | -0.620 | 0.5364 | 0.9196 |
| 1 | 5 | IC6 (AN)-IC11 (FPN) | 72/67 | 0.076 | 0.099 | -0.045 | -0.760 | 0.4485 | 0.9196 |
| 1 | 6 | IC6 (AN)-IC12 (FPN) | 72/67 | 0.137 | 0.137 | -0.004 | -0.084 | 0.9329 | 0.9954 |
| 1 | 7 | IC6 (AN)-IC7 (SMN) | 72/67 | 0.473 | 0.446 | 0.008 | 0.132 | 0.8954 | 0.9954 |
| 1 | 8 | IC6 (AN)-IC10 (VN) | 72/67 | -0.124 | -0.119 | 0.002 | 0.039 | 0.9686 | 0.9954 |
| 1 | 9 | IC4 (DMN)-IC20 (DMN) | 72/67 | 0.053 | 0.091 | -0.046 | -0.779 | 0.4373 | 0.9196 |
| 1 | 10 | IC4 (DMN)-IC5 (DAN) | 72/67 | 0.215 | 0.221 | -0.008 | -0.116 | 0.9081 | 0.9954 |
| 1 | 11 | IC4 (DMN)-IC13 (DAN) | 72/67 | 0.045 | 0.086 | -0.023 | -0.407 | 0.6849 | 0.9877 |
| 1 | 12 | IC4 (DMN)-IC11 (FPN) | 72/67 | 0.075 | 0.027 | 0.046 | 0.933 | 0.3526 | 0.9196 |
| 1 | 13 | IC4 (DMN)-IC12 (FPN) | 72/67 | 0.415 | 0.320 | 0.074 | 1.324 | 0.1879 | 0.9196 |
| 1 | 14 | IC4 (DMN)-IC7 (SMN) | 72/67 | 0.168 | 0.173 | 0.023 | 0.445 | 0.6571 | 0.9877 |
| 1 | 15 | IC4 (DMN)-IC10 (VN) | 72/67 | 0.105 | 0.157 | -0.040 | -0.700 | 0.4852 | 0.9196 |
| 1 | 16 | IC20 (DMN)-IC5 (DAN) | 72/67 | -0.639 | -0.685 | 0.065 | 1.245 | 0.2153 | 0.9196 |
| 1 | 17 | IC20 (DMN)-IC13 (DAN) | 72/67 | -0.698 | -0.732 | 0.020 | 0.368 | 0.7133 | 0.9877 |
| 1 | 18 | IC20 (DMN)-IC11 (FPN) | 72/67 | -0.161 | -0.230 | 0.072 | 1.262 | 0.2090 | 0.9196 |
| 1 | 19 | IC20 (DMN)-IC12 (FPN) | 72/67 | -0.165 | -0.160 | 0.005 | 0.087 | 0.9305 | 0.9954 |
| 1 | 20 | IC20 (DMN)-IC7 (SMN) | 72/67 | -0.130 | -0.205 | 0.069 | 1.095 | 0.2757 | 0.9196 |
| 1 | 21 | IC20 (DMN)-IC10 (VN) | 72/67 | 0.383 | 0.387 | -0.027 | -0.459 | 0.6473 | 0.9877 |
| 1 | 22 | IC5 (DAN)-IC13 (DAN) | 72/67 | 0.642 | 0.667 | 0.007 | 0.131 | 0.8962 | 0.9954 |
| 1 | 23 | IC5 (DAN)-IC11 (FPN) | 72/67 | 0.248 | 0.207 | 0.051 | 0.833 | 0.4065 | 0.9196 |
| 1 | 24 | IC5 (DAN)-IC12 (FPN) | 72/67 | 0.403 | 0.304 | 0.077 | 1.477 | 0.1422 | 0.9196 |
| 1 | 25 | IC5 (DAN)-IC7 (SMN) | 72/67 | 0.123 | 0.193 | -0.044 | -0.775 | 0.4396 | 0.9196 |
| 1 | 26 | IC5 (DAN)-IC10 (VN) | 72/67 | -0.503 | -0.434 | -0.033 | -0.645 | 0.5199 | 0.9196 |
| 1 | 27 | IC13 (DAN)-IC11 (FPN) | 72/67 | 0.335 | 0.349 | 0.002 | 0.041 | 0.9673 | 0.9954 |
| 1 | 28 | IC13 (DAN)-IC12 (FPN) | 72/67 | 0.204 | 0.227 | -0.003 | -0.063 | 0.9500 | 0.9954 |
| 1 | 29 | IC13 (DAN)-IC7 (SMN) | 72/67 | 0.380 | 0.370 | 0.000 | 0.006 | 0.9954 | 0.9954 |
| 1 | 30 | IC13 (DAN)-IC10 (VN) | 72/67 | -0.193 | -0.146 | -0.056 | -1.041 | 0.2998 | 0.9196 |
| 1 | 31 | IC11 (FPN)-IC12 (FPN) | 72/67 | 0.457 | 0.384 | 0.097 | 1.693 | 0.0927 | 0.9196 |
| 1 | 32 | IC11 (FPN)-IC7 (SMN) | 72/67 | 0.039 | 0.081 | -0.020 | -0.374 | 0.7088 | 0.9877 |
| 1 | 33 | IC11 (FPN)-IC10 (VN) | 72/67 | -0.156 | -0.117 | -0.046 | -0.842 | 0.4013 | 0.9196 |
| 1 | 34 | IC12 (FPN)-IC7 (SMN) | 72/67 | 0.063 | 0.127 | -0.056 | -1.076 | 0.2838 | 0.9196 |
| 1 | 35 | IC12 (FPN)-IC10 (VN) | 72/67 | -0.167 | -0.019 | -0.119 | -2.456 | 0.0153 | 0.5526 |
| 1 | 36 | IC7 (SMN)-IC10 (VN) | 72/67 | 0.099 | 0.033 | 0.064 | 1.062 | 0.2900 | 0.9196 |
| 2 | 1 | IC6 (AN)-IC4 (DMN) | 35/48 | 0.014 | 0.021 | 0.037 | 0.480 | 0.6323 | 0.8909 |
| 2 | 2 | IC6 (AN)-IC20 (DMN) | 35/48 | -0.590 | -0.662 | 0.029 | 0.308 | 0.7591 | 0.8909 |
| 2 | 3 | IC6 (AN)-IC5 (DAN) | 35/48 | -0.331 | -0.289 | 0.001 | 0.016 | 0.9876 | 0.9931 |
| 2 | 4 | IC6 (AN)-IC13 (DAN) | 35/48 | 0.731 | 0.784 | -0.064 | -0.807 | 0.4221 | 0.8909 |
| 2 | 5 | IC6 (AN)-IC11 (FPN) | 35/48 | -0.209 | -0.127 | -0.055 | -0.675 | 0.5017 | 0.8909 |
| 2 | 6 | IC6 (AN)-IC12 (FPN) | 35/48 | -0.249 | -0.280 | 0.043 | 0.646 | 0.5205 | 0.8909 |
| 2 | 7 | IC6 (AN)-IC7 (SMN) | 35/48 | 0.861 | 0.749 | 0.115 | 1.492 | 0.1397 | 0.8909 |
| 2 | 8 | IC6 (AN)-IC10 (VN) | 35/48 | 0.504 | 0.444 | 0.059 | 0.886 | 0.3781 | 0.8909 |
| 2 | 9 | IC4 (DMN)-IC20 (DMN) | 35/48 | 0.099 | 0.166 | -0.061 | -0.622 | 0.5355 | 0.8909 |
| 2 | 10 | IC4 (DMN)-IC5 (DAN) | 35/48 | 0.230 | 0.279 | -0.032 | -0.411 | 0.6819 | 0.8909 |
| 2 | 11 | IC4 (DMN)-IC13 (DAN) | 35/48 | -0.047 | 0.005 | -0.050 | -0.650 | 0.5175 | 0.8909 |
| 2 | 12 | IC4 (DMN)-IC11 (FPN) | 35/48 | -0.002 | 0.075 | -0.062 | -0.851 | 0.3974 | 0.8909 |
| 2 | 13 | IC4 (DMN)-IC12 (FPN) | 35/48 | 0.209 | 0.278 | -0.042 | -0.455 | 0.6501 | 0.8909 |
| 2 | 14 | IC4 (DMN)-IC7 (SMN) | 35/48 | 0.126 | 0.186 | -0.055 | -0.674 | 0.5023 | 0.8909 |
| 2 | 15 | IC4 (DMN)-IC10 (VN) | 35/48 | 0.113 | 0.198 | -0.100 | -1.109 | 0.2708 | 0.8909 |
| 2 | 16 | IC20 (DMN)-IC5 (DAN) | 35/48 | 0.016 | 0.008 | 0.038 | 0.516 | 0.6074 | 0.8909 |
| 2 | 17 | IC20 (DMN)-IC13 (DAN) | 35/48 | -0.586 | -0.548 | -0.073 | -0.781 | 0.4374 | 0.8909 |
| 2 | 18 | IC20 (DMN)-IC11 (FPN) | 35/48 | 0.004 | 0.013 | -0.008 | -0.093 | 0.9258 | 0.9931 |
| 2 | 19 | IC20 (DMN)-IC12 (FPN) | 35/48 | 0.135 | 0.253 | -0.117 | -1.653 | 0.1024 | 0.8909 |
| 2 | 20 | IC20 (DMN)-IC7 (SMN) | 35/48 | -0.344 | -0.321 | -0.045 | -0.527 | 0.5999 | 0.8909 |
| 2 | 21 | IC20 (DMN)-IC10 (VN) | 35/48 | -0.273 | -0.176 | -0.118 | -1.571 | 0.1203 | 0.8909 |
| 2 | 22 | IC5 (DAN)-IC13 (DAN) | 35/48 | -0.564 | -0.602 | 0.052 | 0.684 | 0.4960 | 0.8909 |
| 2 | 23 | IC5 (DAN)-IC11 (FPN) | 35/48 | 0.188 | 0.160 | 0.003 | 0.037 | 0.9707 | 0.9931 |
| 2 | 24 | IC5 (DAN)-IC12 (FPN) | 35/48 | 0.432 | 0.472 | -0.043 | -0.570 | 0.5706 | 0.8909 |
| 2 | 25 | IC5 (DAN)-IC7 (SMN) | 35/48 | -0.536 | -0.463 | -0.039 | -0.475 | 0.6359 | 0.8909 |
| 2 | 26 | IC5 (DAN)-IC10 (VN) | 35/48 | -0.577 | -0.603 | 0.001 | 0.009 | 0.9931 | 0.9931 |
| 2 | 27 | IC13 (DAN)-IC11 (FPN) | 35/48 | -0.118 | -0.122 | 0.025 | 0.322 | 0.7483 | 0.8909 |
| 2 | 28 | IC13 (DAN)-IC12 (FPN) | 35/48 | -0.448 | -0.472 | 0.014 | 0.207 | 0.8366 | 0.9412 |
| 2 | 29 | IC13 (DAN)-IC7 (SMN) | 35/48 | 0.867 | 0.869 | -0.033 | -0.417 | 0.6777 | 0.8909 |
| 2 | 30 | IC13 (DAN)-IC10 (VN) | 35/48 | 0.811 | 0.866 | -0.026 | -0.299 | 0.7657 | 0.8909 |
| 2 | 31 | IC11 (FPN)-IC12 (FPN) | 35/48 | 0.548 | 0.485 | 0.053 | 0.625 | 0.5336 | 0.8909 |
| 2 | 32 | IC11 (FPN)-IC7 (SMN) | 35/48 | -0.186 | -0.084 | -0.054 | -0.720 | 0.4736 | 0.8909 |
| 2 | 33 | IC11 (FPN)-IC10 (VN) | 35/48 | -0.205 | -0.224 | 0.022 | 0.297 | 0.7671 | 0.8909 |
| 2 | 34 | IC12 (FPN)-IC7 (SMN) | 35/48 | -0.338 | -0.279 | -0.049 | -0.755 | 0.4525 | 0.8909 |
| 2 | 35 | IC12 (FPN)-IC10 (VN) | 35/48 | -0.434 | -0.394 | -0.059 | -0.780 | 0.4378 | 0.8909 |
| 2 | 36 | IC7 (SMN)-IC10 (VN) | 35/48 | 0.679 | 0.610 | 0.067 | 0.877 | 0.3830 | 0.8909 |
| 3 | 1 | IC6 (AN)-IC4 (DMN) | 120/105 | -0.041 | -0.005 | -0.039 | -0.939 | 0.3490 | 0.9099 |
| 3 | 2 | IC6 (AN)-IC20 (DMN) | 120/105 | -0.357 | -0.396 | 0.049 | 1.145 | 0.2536 | 0.9099 |
| 3 | 3 | IC6 (AN)-IC5 (DAN) | 120/105 | 0.018 | 0.055 | -0.030 | -0.689 | 0.4913 | 0.9099 |
| 3 | 4 | IC6 (AN)-IC13 (DAN) | 120/105 | 0.250 | 0.267 | -0.019 | -0.455 | 0.6493 | 0.9099 |
| 3 | 5 | IC6 (AN)-IC11 (FPN) | 120/105 | -0.107 | -0.137 | 0.028 | 0.710 | 0.4784 | 0.9099 |
| 3 | 6 | IC6 (AN)-IC12 (FPN) | 120/105 | -0.080 | -0.044 | -0.033 | -0.791 | 0.4300 | 0.9099 |
| 3 | 7 | IC6 (AN)-IC7 (SMN) | 120/105 | 0.355 | 0.348 | 0.007 | 0.154 | 0.8776 | 0.9099 |
| 3 | 8 | IC6 (AN)-IC10 (VN) | 120/105 | 0.102 | 0.145 | -0.039 | -0.953 | 0.3418 | 0.9099 |
| 3 | 9 | IC4 (DMN)-IC20 (DMN) | 120/105 | 0.152 | 0.209 | -0.063 | -1.398 | 0.1636 | 0.9099 |
| 3 | 10 | IC4 (DMN)-IC5 (DAN) | 120/105 | 0.169 | 0.170 | -0.006 | -0.133 | 0.8943 | 0.9099 |
| 3 | 11 | IC4 (DMN)-IC13 (DAN) | 120/105 | 0.012 | -0.007 | 0.018 | 0.401 | 0.6891 | 0.9099 |
| 3 | 12 | IC4 (DMN)-IC11 (FPN) | 120/105 | -0.072 | -0.014 | -0.056 | -1.347 | 0.1793 | 0.9099 |
| 3 | 13 | IC4 (DMN)-IC12 (FPN) | 120/105 | 0.291 | 0.324 | -0.026 | -0.576 | 0.5653 | 0.9099 |
| 3 | 14 | IC4 (DMN)-IC7 (SMN) | 120/105 | 0.190 | 0.239 | -0.051 | -1.229 | 0.2203 | 0.9099 |
| 3 | 15 | IC4 (DMN)-IC10 (VN) | 120/105 | 0.225 | 0.203 | 0.034 | 0.790 | 0.4304 | 0.9099 |
| 3 | 16 | IC20 (DMN)-IC5 (DAN) | 120/105 | -0.265 | -0.281 | 0.011 | 0.235 | 0.8148 | 0.9099 |
| 3 | 17 | IC20 (DMN)-IC13 (DAN) | 120/105 | -0.419 | -0.395 | -0.023 | -0.520 | 0.6036 | 0.9099 |
| 3 | 18 | IC20 (DMN)-IC11 (FPN) | 120/105 | 0.008 | -0.012 | 0.018 | 0.371 | 0.7109 | 0.9099 |
| 3 | 19 | IC20 (DMN)-IC12 (FPN) | 120/105 | 0.114 | 0.099 | 0.014 | 0.313 | 0.7548 | 0.9099 |
| 3 | 20 | IC20 (DMN)-IC7 (SMN) | 120/105 | 0.112 | 0.031 | 0.096 | 2.268 | 0.0243 | 0.8062 |
| 3 | 21 | IC20 (DMN)-IC10 (VN) | 120/105 | 0.054 | 0.048 | -0.006 | -0.113 | 0.9099 | 0.9099 |
| 3 | 22 | IC5 (DAN)-IC13 (DAN) | 120/105 | 0.155 | 0.085 | 0.087 | 2.018 | 0.0448 | 0.8062 |
| 3 | 23 | IC5 (DAN)-IC11 (FPN) | 120/105 | 0.034 | 0.046 | -0.011 | -0.250 | 0.8028 | 0.9099 |
| 3 | 24 | IC5 (DAN)-IC12 (FPN) | 120/105 | 0.189 | 0.151 | 0.038 | 0.837 | 0.4036 | 0.9099 |
| 3 | 25 | IC5 (DAN)-IC7 (SMN) | 120/105 | -0.170 | -0.116 | -0.050 | -1.159 | 0.2476 | 0.9099 |
| 3 | 26 | IC5 (DAN)-IC10 (VN) | 120/105 | -0.223 | -0.242 | 0.034 | 0.693 | 0.4891 | 0.9099 |
| 3 | 27 | IC13 (DAN)-IC11 (FPN) | 120/105 | 0.145 | 0.192 | -0.038 | -0.829 | 0.4079 | 0.9099 |
| 3 | 28 | IC13 (DAN)-IC12 (FPN) | 120/105 | -0.082 | -0.022 | -0.059 | -1.540 | 0.1250 | 0.9099 |
| 3 | 29 | IC13 (DAN)-IC7 (SMN) | 120/105 | 0.198 | 0.194 | -0.012 | -0.305 | 0.7605 | 0.9099 |
| 3 | 30 | IC13 (DAN)-IC10 (VN) | 120/105 | 0.273 | 0.281 | -0.009 | -0.188 | 0.8513 | 0.9099 |
| 3 | 31 | IC11 (FPN)-IC12 (FPN) | 120/105 | 0.383 | 0.376 | 0.007 | 0.147 | 0.8830 | 0.9099 |
| 3 | 32 | IC11 (FPN)-IC7 (SMN) | 120/105 | -0.046 | -0.074 | 0.027 | 0.652 | 0.5151 | 0.9099 |
| 3 | 33 | IC11 (FPN)-IC10 (VN) | 120/105 | -0.061 | -0.060 | 0.009 | 0.220 | 0.8264 | 0.9099 |
| 3 | 34 | IC12 (FPN)-IC7 (SMN) | 120/105 | 0.031 | 0.029 | 0.005 | 0.131 | 0.8961 | 0.9099 |
| 3 | 35 | IC12 (FPN)-IC10 (VN) | 120/105 | -0.080 | -0.053 | -0.016 | -0.350 | 0.7268 | 0.9099 |
| 3 | 36 | IC7 (SMN)-IC10 (VN) | 120/105 | 0.181 | 0.147 | 0.025 | 0.541 | 0.5892 | 0.9099 |
| 4 | 1 | IC6 (AN)-IC4 (DMN) | 59/41 | 0.178 | 0.141 | 0.053 | 0.814 | 0.4174 | 0.9096 |
| 4 | 2 | IC6 (AN)-IC20 (DMN) | 59/41 | -0.570 | -0.558 | -0.025 | -0.343 | 0.7327 | 0.9096 |
| 4 | 3 | IC6 (AN)-IC5 (DAN) | 59/41 | 0.357 | 0.278 | 0.062 | 0.925 | 0.3574 | 0.9096 |
| 4 | 4 | IC6 (AN)-IC13 (DAN) | 59/41 | 0.470 | 0.531 | -0.025 | -0.347 | 0.7294 | 0.9096 |
| 4 | 5 | IC6 (AN)-IC11 (FPN) | 59/41 | -0.251 | -0.325 | 0.085 | 1.162 | 0.2482 | 0.9096 |
| 4 | 6 | IC6 (AN)-IC12 (FPN) | 59/41 | -0.138 | -0.171 | 0.019 | 0.269 | 0.7884 | 0.9460 |
| 4 | 7 | IC6 (AN)-IC7 (SMN) | 59/41 | 0.585 | 0.560 | 0.057 | 0.788 | 0.4326 | 0.9096 |
| 4 | 8 | IC6 (AN)-IC10 (VN) | 59/41 | 0.322 | 0.364 | -0.047 | -0.697 | 0.4874 | 0.9096 |
| 4 | 9 | IC4 (DMN)-IC20 (DMN) | 59/41 | -0.143 | -0.028 | -0.106 | -1.534 | 0.1283 | 0.9096 |
| 4 | 10 | IC4 (DMN)-IC5 (DAN) | 59/41 | 0.368 | 0.382 | -0.029 | -0.346 | 0.7304 | 0.9096 |
| 4 | 11 | IC4 (DMN)-IC13 (DAN) | 59/41 | 0.282 | 0.165 | 0.100 | 1.204 | 0.2318 | 0.9096 |
| 4 | 12 | IC4 (DMN)-IC11 (FPN) | 59/41 | -0.244 | -0.080 | -0.151 | -1.879 | 0.0633 | 0.9096 |
| 4 | 13 | IC4 (DMN)-IC12 (FPN) | 59/41 | 0.100 | 0.229 | -0.135 | -1.827 | 0.0709 | 0.9096 |
| 4 | 14 | IC4 (DMN)-IC7 (SMN) | 59/41 | 0.435 | 0.311 | 0.112 | 1.344 | 0.1823 | 0.9096 |
| 4 | 15 | IC4 (DMN)-IC10 (VN) | 59/41 | 0.360 | 0.268 | 0.067 | 0.792 | 0.4305 | 0.9096 |
| 4 | 16 | IC20 (DMN)-IC5 (DAN) | 59/41 | -0.573 | -0.583 | 0.041 | 0.628 | 0.5315 | 0.9096 |
| 4 | 17 | IC20 (DMN)-IC13 (DAN) | 59/41 | -0.687 | -0.750 | 0.046 | 0.637 | 0.5256 | 0.9096 |
| 4 | 18 | IC20 (DMN)-IC11 (FPN) | 59/41 | 0.215 | 0.164 | 0.070 | 0.947 | 0.3459 | 0.9096 |
| 4 | 19 | IC20 (DMN)-IC12 (FPN) | 59/41 | 0.235 | 0.201 | 0.049 | 0.728 | 0.4682 | 0.9096 |
| 4 | 20 | IC20 (DMN)-IC7 (SMN) | 59/41 | -0.168 | -0.256 | 0.076 | 1.152 | 0.2523 | 0.9096 |
| 4 | 21 | IC20 (DMN)-IC10 (VN) | 59/41 | -0.377 | -0.391 | 0.011 | 0.154 | 0.8783 | 0.9533 |
| 4 | 22 | IC5 (DAN)-IC13 (DAN) | 59/41 | 0.482 | 0.397 | 0.060 | 1.021 | 0.3098 | 0.9096 |
| 4 | 23 | IC5 (DAN)-IC11 (FPN) | 59/41 | -0.161 | -0.189 | 0.040 | 0.673 | 0.5025 | 0.9096 |
| 4 | 24 | IC5 (DAN)-IC12 (FPN) | 59/41 | -0.017 | 0.013 | -0.055 | -0.702 | 0.4841 | 0.9096 |
| 4 | 25 | IC5 (DAN)-IC7 (SMN) | 59/41 | 0.248 | 0.253 | -0.037 | -0.538 | 0.5920 | 0.9096 |
| 4 | 26 | IC5 (DAN)-IC10 (VN) | 59/41 | 0.216 | 0.126 | 0.082 | 1.161 | 0.2487 | 0.9096 |
| 4 | 27 | IC13 (DAN)-IC11 (FPN) | 59/41 | -0.108 | -0.101 | -0.009 | -0.120 | 0.9045 | 0.9533 |
| 4 | 28 | IC13 (DAN)-IC12 (FPN) | 59/41 | -0.179 | -0.237 | 0.043 | 0.719 | 0.4738 | 0.9096 |
| 4 | 29 | IC13 (DAN)-IC7 (SMN) | 59/41 | 0.494 | 0.474 | 0.035 | 0.407 | 0.6846 | 0.9096 |
| 4 | 30 | IC13 (DAN)-IC10 (VN) | 59/41 | 0.652 | 0.645 | 0.017 | 0.199 | 0.8426 | 0.9533 |
| 4 | 31 | IC11 (FPN)-IC12 (FPN) | 59/41 | 0.431 | 0.418 | 0.038 | 0.483 | 0.6300 | 0.9096 |
| 4 | 32 | IC11 (FPN)-IC7 (SMN) | 59/41 | -0.255 | -0.239 | -0.004 | -0.053 | 0.9577 | 0.9577 |
| 4 | 33 | IC11 (FPN)-IC10 (VN) | 59/41 | -0.250 | -0.233 | -0.007 | -0.092 | 0.9269 | 0.9533 |
| 4 | 34 | IC12 (FPN)-IC7 (SMN) | 59/41 | -0.088 | -0.060 | -0.040 | -0.571 | 0.5692 | 0.9096 |
| 4 | 35 | IC12 (FPN)-IC10 (VN) | 59/41 | -0.209 | -0.261 | 0.027 | 0.435 | 0.6647 | 0.9096 |
| 4 | 36 | IC7 (SMN)-IC10 (VN) | 59/41 | 0.341 | 0.346 | 0.009 | 0.094 | 0.9253 | 0.9533 |
| 5 | 1 | IC6 (AN)-IC4 (DMN) | 72/70 | -0.297 | -0.289 | 0.011 | 0.190 | 0.8498 | 0.9648 |
| 5 | 2 | IC6 (AN)-IC20 (DMN) | 72/70 | -0.734 | -0.782 | 0.065 | 1.244 | 0.2158 | 0.7552 |
| 5 | 3 | IC6 (AN)-IC5 (DAN) | 72/70 | 0.358 | 0.323 | 0.036 | 0.689 | 0.4917 | 0.8207 |
| 5 | 4 | IC6 (AN)-IC13 (DAN) | 72/70 | 0.647 | 0.624 | 0.021 | 0.408 | 0.6836 | 0.8884 |
| 5 | 5 | IC6 (AN)-IC11 (FPN) | 72/70 | -0.230 | -0.212 | -0.016 | -0.266 | 0.7909 | 0.9648 |
| 5 | 6 | IC6 (AN)-IC12 (FPN) | 72/70 | -0.380 | -0.319 | -0.054 | -1.045 | 0.2981 | 0.8207 |
| 5 | 7 | IC6 (AN)-IC7 (SMN) | 72/70 | 0.589 | 0.517 | 0.083 | 1.255 | 0.2117 | 0.7552 |
| 5 | 8 | IC6 (AN)-IC10 (VN) | 72/70 | -0.048 | -0.038 | 0.006 | 0.112 | 0.9112 | 0.9648 |
| 5 | 9 | IC4 (DMN)-IC20 (DMN) | 72/70 | 0.385 | 0.410 | -0.043 | -0.743 | 0.4587 | 0.8207 |
| 5 | 10 | IC4 (DMN)-IC5 (DAN) | 72/70 | -0.097 | -0.134 | 0.022 | 0.398 | 0.6910 | 0.8884 |
| 5 | 11 | IC4 (DMN)-IC13 (DAN) | 72/70 | -0.224 | -0.230 | 0.031 | 0.564 | 0.5734 | 0.8258 |
| 5 | 12 | IC4 (DMN)-IC11 (FPN) | 72/70 | 0.059 | 0.118 | -0.067 | -1.232 | 0.2202 | 0.7552 |
| 5 | 13 | IC4 (DMN)-IC12 (FPN) | 72/70 | 0.364 | 0.446 | -0.092 | -1.699 | 0.0916 | 0.7552 |
| 5 | 14 | IC4 (DMN)-IC7 (SMN) | 72/70 | 0.021 | -0.011 | 0.039 | 0.657 | 0.5122 | 0.8207 |
| 5 | 15 | IC4 (DMN)-IC10 (VN) | 72/70 | 0.234 | 0.337 | -0.097 | -1.723 | 0.0872 | 0.7552 |
| 5 | 16 | IC20 (DMN)-IC5 (DAN) | 72/70 | -0.521 | -0.534 | -0.009 | -0.150 | 0.8812 | 0.9648 |
| 5 | 17 | IC20 (DMN)-IC13 (DAN) | 72/70 | -0.784 | -0.735 | -0.034 | -0.638 | 0.5243 | 0.8207 |
| 5 | 18 | IC20 (DMN)-IC11 (FPN) | 72/70 | 0.102 | 0.185 | -0.067 | -1.204 | 0.2307 | 0.7552 |
| 5 | 19 | IC20 (DMN)-IC12 (FPN) | 72/70 | 0.381 | 0.365 | 0.006 | 0.119 | 0.9053 | 0.9648 |
| 5 | 20 | IC20 (DMN)-IC7 (SMN) | 72/70 | -0.269 | -0.327 | 0.069 | 1.287 | 0.2004 | 0.7552 |
| 5 | 21 | IC20 (DMN)-IC10 (VN) | 72/70 | 0.222 | 0.246 | -0.038 | -0.594 | 0.5534 | 0.8258 |
| 5 | 22 | IC5 (DAN)-IC13 (DAN) | 72/70 | 0.457 | 0.423 | 0.044 | 0.905 | 0.3669 | 0.8207 |
| 5 | 23 | IC5 (DAN)-IC11 (FPN) | 72/70 | -0.144 | -0.113 | -0.076 | -1.332 | 0.1850 | 0.7552 |
| 5 | 24 | IC5 (DAN)-IC12 (FPN) | 72/70 | -0.150 | -0.110 | -0.077 | -1.424 | 0.1568 | 0.7552 |
| 5 | 25 | IC5 (DAN)-IC7 (SMN) | 72/70 | 0.098 | 0.174 | -0.077 | -1.442 | 0.1515 | 0.7552 |
| 5 | 26 | IC5 (DAN)-IC10 (VN) | 72/70 | -0.343 | -0.340 | -0.002 | -0.031 | 0.9750 | 0.9750 |
| 5 | 27 | IC13 (DAN)-IC11 (FPN) | 72/70 | 0.027 | 0.014 | -0.006 | -0.115 | 0.9086 | 0.9648 |
| 5 | 28 | IC13 (DAN)-IC12 (FPN) | 72/70 | -0.297 | -0.242 | -0.047 | -0.869 | 0.3863 | 0.8207 |
| 5 | 29 | IC13 (DAN)-IC7 (SMN) | 72/70 | 0.462 | 0.502 | -0.037 | -0.641 | 0.5224 | 0.8207 |
| 5 | 30 | IC13 (DAN)-IC10 (VN) | 72/70 | -0.008 | 0.032 | -0.043 | -0.724 | 0.4704 | 0.8207 |
| 5 | 31 | IC11 (FPN)-IC12 (FPN) | 72/70 | 0.450 | 0.442 | 0.026 | 0.428 | 0.6692 | 0.8884 |
| 5 | 32 | IC11 (FPN)-IC7 (SMN) | 72/70 | -0.124 | -0.133 | 0.003 | 0.066 | 0.9474 | 0.9744 |
| 5 | 33 | IC11 (FPN)-IC10 (VN) | 72/70 | -0.014 | 0.034 | -0.043 | -0.729 | 0.4673 | 0.8207 |
| 5 | 34 | IC12 (FPN)-IC7 (SMN) | 72/70 | -0.157 | -0.194 | 0.063 | 1.302 | 0.1951 | 0.7552 |
| 5 | 35 | IC12 (FPN)-IC10 (VN) | 72/70 | 0.072 | 0.123 | -0.048 | -0.767 | 0.4445 | 0.8207 |
| 5 | 36 | IC7 (SMN)-IC10 (VN) | 72/70 | 0.092 | 0.060 | 0.061 | 1.136 | 0.2579 | 0.7736 |
| 6 | 1 | IC6 (AN)-IC4 (DMN) | 53/73 | 0.152 | 0.199 | -0.046 | -0.815 | 0.4169 | 0.8551 |
| 6 | 2 | IC6 (AN)-IC20 (DMN) | 53/73 | -0.498 | -0.551 | 0.054 | 0.887 | 0.3767 | 0.8551 |
| 6 | 3 | IC6 (AN)-IC5 (DAN) | 53/73 | 0.079 | 0.002 | 0.089 | 1.556 | 0.1224 | 0.5841 |
| 6 | 4 | IC6 (AN)-IC13 (DAN) | 53/73 | 0.585 | 0.605 | -0.014 | -0.258 | 0.7965 | 0.9074 |
| 6 | 5 | IC6 (AN)-IC11 (FPN) | 53/73 | 0.187 | 0.220 | -0.032 | -0.564 | 0.5738 | 0.8980 |
| 6 | 6 | IC6 (AN)-IC12 (FPN) | 53/73 | 0.187 | 0.180 | -0.004 | -0.063 | 0.9495 | 0.9854 |
| 6 | 7 | IC6 (AN)-IC7 (SMN) | 53/73 | 0.573 | 0.577 | -0.002 | -0.032 | 0.9746 | 0.9854 |
| 6 | 8 | IC6 (AN)-IC10 (VN) | 53/73 | 0.343 | 0.446 | -0.117 | -2.373 | 0.0193 | 0.3466 |
| 6 | 9 | IC4 (DMN)-IC20 (DMN) | 53/73 | -0.025 | -0.056 | 0.042 | 0.756 | 0.4513 | 0.8551 |
| 6 | 10 | IC4 (DMN)-IC5 (DAN) | 53/73 | 0.302 | 0.281 | 0.017 | 0.273 | 0.7857 | 0.9074 |
| 6 | 11 | IC4 (DMN)-IC13 (DAN) | 53/73 | 0.280 | 0.305 | -0.050 | -0.802 | 0.4244 | 0.8551 |
| 6 | 12 | IC4 (DMN)-IC11 (FPN) | 53/73 | 0.219 | 0.237 | -0.031 | -0.596 | 0.5522 | 0.8980 |
| 6 | 13 | IC4 (DMN)-IC12 (FPN) | 53/73 | 0.468 | 0.557 | -0.096 | -1.621 | 0.1077 | 0.5841 |
| 6 | 14 | IC4 (DMN)-IC7 (SMN) | 53/73 | 0.397 | 0.492 | -0.103 | -1.740 | 0.0845 | 0.5841 |
| 6 | 15 | IC4 (DMN)-IC10 (VN) | 53/73 | 0.446 | 0.461 | -0.021 | -0.335 | 0.7383 | 0.9074 |
| 6 | 16 | IC20 (DMN)-IC5 (DAN) | 53/73 | -0.242 | -0.263 | 0.022 | 0.320 | 0.7492 | 0.9074 |
| 6 | 17 | IC20 (DMN)-IC13 (DAN) | 53/73 | -0.582 | -0.567 | -0.031 | -0.503 | 0.6159 | 0.9074 |
| 6 | 18 | IC20 (DMN)-IC11 (FPN) | 53/73 | -0.210 | -0.202 | -0.026 | -0.441 | 0.6600 | 0.9074 |
| 6 | 19 | IC20 (DMN)-IC12 (FPN) | 53/73 | -0.057 | -0.070 | 0.009 | 0.141 | 0.8883 | 0.9690 |
| 6 | 20 | IC20 (DMN)-IC7 (SMN) | 53/73 | -0.152 | -0.264 | 0.100 | 1.525 | 0.1298 | 0.5841 |
| 6 | 21 | IC20 (DMN)-IC10 (VN) | 53/73 | -0.213 | -0.260 | 0.066 | 0.930 | 0.3543 | 0.8551 |
| 6 | 22 | IC5 (DAN)-IC13 (DAN) | 53/73 | 0.158 | 0.066 | 0.095 | 1.316 | 0.1907 | 0.7568 |
| 6 | 23 | IC5 (DAN)-IC11 (FPN) | 53/73 | 0.087 | 0.136 | -0.029 | -0.406 | 0.6853 | 0.9074 |
| 6 | 24 | IC5 (DAN)-IC12 (FPN) | 53/73 | 0.162 | 0.177 | -0.001 | -0.018 | 0.9854 | 0.9854 |
| 6 | 25 | IC5 (DAN)-IC7 (SMN) | 53/73 | 0.016 | 0.040 | -0.026 | -0.464 | 0.6434 | 0.9074 |
| 6 | 26 | IC5 (DAN)-IC10 (VN) | 53/73 | -0.073 | -0.166 | 0.083 | 1.180 | 0.2404 | 0.7568 |
| 6 | 27 | IC13 (DAN)-IC11 (FPN) | 53/73 | 0.383 | 0.356 | 0.042 | 0.709 | 0.4796 | 0.8605 |
| 6 | 28 | IC13 (DAN)-IC12 (FPN) | 53/73 | 0.259 | 0.210 | 0.038 | 0.673 | 0.5020 | 0.8605 |
| 6 | 29 | IC13 (DAN)-IC7 (SMN) | 53/73 | 0.559 | 0.632 | -0.073 | -1.131 | 0.2601 | 0.7568 |
| 6 | 30 | IC13 (DAN)-IC10 (VN) | 53/73 | 0.566 | 0.664 | -0.119 | -1.779 | 0.0777 | 0.5841 |
| 6 | 31 | IC11 (FPN)-IC12 (FPN) | 53/73 | 0.533 | 0.454 | 0.072 | 1.175 | 0.2425 | 0.7568 |
| 6 | 32 | IC11 (FPN)-IC7 (SMN) | 53/73 | 0.238 | 0.251 | -0.013 | -0.245 | 0.8066 | 0.9074 |
| 6 | 33 | IC11 (FPN)-IC10 (VN) | 53/73 | 0.259 | 0.145 | 0.119 | 2.393 | 0.0183 | 0.3466 |
| 6 | 34 | IC12 (FPN)-IC7 (SMN) | 53/73 | 0.321 | 0.281 | 0.054 | 1.101 | 0.2733 | 0.7568 |
| 6 | 35 | IC12 (FPN)-IC10 (VN) | 53/73 | 0.271 | 0.222 | 0.040 | 0.766 | 0.4452 | 0.8551 |
| 6 | 36 | IC7 (SMN)-IC10 (VN) | 53/73 | 0.376 | 0.481 | -0.110 | -1.790 | 0.0759 | 0.5841 |

*Note.* Analyses included participants with an available subject-level connectivity pattern for the corresponding state. Unadjusted group means are shown for description; adjusted beta and t values were obtained from linear models controlling for mean framewise displacement, age, sex, and years of education. Adjusted beta values represent LBE minus NLB. FDR correction was applied separately across the 36 edges within each state. No edge survived FDR correction. Network abbreviations are defined in Supplementary Table S5.

## Supplementary Figures

**Supplementary Figure S1.** Complementary validation of the number of dynamic functional connectivity states. The elbow criterion indicated a marked inflection at K = 6; the gap statistic reached its maximum at K = 8; the mean silhouette coefficient reached its maximum at K = 6; and repeated split-half reproducibility, summarized by the adjusted Rand index (ARI), reached its maximum at K = 6. The six-state solution was retained based on convergence of the elbow, silhouette, and split-half criteria together with parsimony.

**Supplementary Figure S2.** Within-state consistency of the six dFNC states. Distributions show Pearson correlations between individual sliding-window dFNC patterns and the centroid of the state to which each window was assigned. State 2 showed the highest mean consistency, State 3 showed the lowest consistency, and State 6 showed moderate-to-high consistency.

**Supplementary Figure S3.** Between-state separation of the six group-level centroids. (A) Pairwise Pearson correlations between vectorized state centroids. (B) Pairwise Euclidean distances between state centroids. Lower off-diagonal correlations and larger distances indicate greater separation.

**Supplementary Figure S4.** Cross-subject reproducibility of the six dFNC states. Participant-specific mean state patterns were correlated with the corresponding group-level centroid for participants contributing at least three windows to that state. State 6 showed a mean subject-to-centroid correlation of 0.700 (SD = 0.113).

**Supplementary Figure S5.** Network-level characterization of the six dFNC state centroids. (A) Mean Fisher z-transformed within- and between-network connectivity values for all six state centroids. (B) Focused profile of State 6, showing relatively strong AN-SMN, FPN-FPN, SMN-VN, and AN-VN connectivity together with comparatively weak DMN involvement. AN, auditory network; DMN, default mode network; DAN, dorsal attention network; FPN, frontoparietal network; SMN, somatomotor network; VN, visual network.

**Supplementary Figure S6.** Correspondence between the original 30-TR and sensitivity-analysis 20-TR state centroids. Rows represent original 30-TR states and columns represent 20-TR states reordered according to the optimal one-to-one matching. Black boxes identify matched pairs. Matched correlations ranged from r = 0.993 to r = 0.999 (mean r = 0.996), and original 30-TR State 6 matched 20-TR State 3 (r = 0.998).

**Supplementary Figure S7.** Replication of the principal temporal group differences and association with optimism using the 20-TR window. The original 30-TR State 6 corresponded to 20-TR State 3. (A) The LBE group showed a lower fraction of time (FT) in the matched state than the NLB group (FDR-corrected p < 0.001). (B) The LBE group showed a lower mean dwell time (MDT) in the matched state than the NLB group (FDR-corrected p = 0.0070). Group comparisons were adjusted for mean framewise displacement, age, sex, and education, and p values were FDR-corrected across the six states separately for FT and MDT. (C) MDT in the matched state was positively associated with optimism scores after residualizing both variables for the same covariates (ρ = 0.128, uncorrected p = 0.0277).
